# Phytochemical Profiling and Anti‐Inflammatory Effects of Aerial and Underground Parts of *Apium graveolens var. rapaceum* (Celeriac): Potential Health Benefits of Discarded Aerial Parts

**DOI:** 10.1002/fsn3.71011

**Published:** 2025-10-03

**Authors:** Sang A. Kwak, Jane J. Lee, Ju Hong Park, Nami Joo

**Affiliations:** ^1^ Department of Food and Nutrition College of Human Ecology, Sookmyung Women's University Seoul Republic of Korea; ^2^ Department of Convergence IT Engineering Pohang University of Science and Technology (POSTECH) Pohang Republic of Korea

**Keywords:** anti‐inflammatory, *apium graveolens var. rapaceum*, celeriac, functional foods, phytochemicals

## Abstract

*Apium graveolens* var. *rapaceum* *(celeriac*), a root vegetable cultivated as a medicinal plant since ancient times, has gained attention due to its nutritional and health benefits. The aerial parts of celeriac (APC) are rich in compounds such as apigenin, known for its potent anti‐inflammatory properties. However, APC is often discarded due to its tough texture and bitter taste. To assess the health benefits and explore the potential of both the APC and underground parts of celeriac (UPC) as functional food ingredients, their phytochemical profiles and anti‐inflammatory effects were evaluated. In particular, this study aimed to investigate the feasibility of utilizing the often‐discarded APC as a functional food by thoroughly analyzing the composition of the entire celeriac plant. Phytochemical analysis of APC and UPC revealed 31 compounds, including 9 hydroxycinnamic acids, 2 hydroxycoumarins, 1 flavonol, 8 flavones, 8 furanocoumarins, and 3 phthalides. APC contained higher levels of compounds providing anti‐inflammatory effects and health benefits than UPC. When evaluating their anti‐inflammatory effects in LPS‐induced RAW264.7 macrophages, ethanolic extracts of APC (APCE) and UPC (UPCE) reduced NO and PGE_2_ production in a concentration‐dependent manner (*p* < 0.05), decreased mRNA expression levels of iNOS and COX‐2 (*p* < 0.05), and suppressed mRNA levels of pro‐inflammatory cytokines (TNF‐α, IL‐6, IL‐1β). Notably, APCE demonstrated strong anti‐inflammatory effects at higher concentrations (0.5 mg/mL, *p* < 0.05). This study confirms the potential of currently discarded APC as functional food ingredients and pharmacological substances, suggesting they could offer health benefits.

## Graphical abstract

*Apium graveolens var. rapaceum* (celeriac) was analyzed to compare the phytochemical composition and anti‐inflammatory effects of its aerial (APC) and underground (UPC) parts. APC contained higher levels of phytochemical compounds and showed stronger anti‐inflammatory activity in LPS‐induced RAW264.7 macrophages. These findings suggest the potential of APC, typically discarded, as a functional food ingredient.

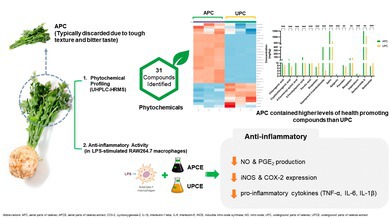

## 1 Introduction

*Apium graveolens var. rapaceum* (celeriac), a variety of celery from the Apiaceae family, is cultivated worldwide for its edible roots, stems, and leaves (Kosson et al. 2014; Rana and Yadav 2017). Traditionally, celeriac has been widely used in both culinary applications and traditional medicine (Kesbiç 2023). Today, it is primarily valued for its edible root, which has a distinctive flavor of celery (Farooqi et al. 2006; Rana and Yadav 2017). Although it is commonly referred to as a root, it is botanically classified as a tuber that can grow up to 15 cm in diameter (Hadley and Fordham 2003). Celeriac plants can reach heights of 60–90 cm and are distinguished from common celery varieties by their darker green, deeply lobed leaves. Celeriac is nutritionally rich in vitamins and minerals, including Fe, Cu, Zn, Mn, and Ni. Notably, the aerial parts of celeriac (APC) contain higher levels of vitamin C than the underground parts (UPC) (Godlewska et al. 2020). Despite its nutritional value, APC is often discarded due to its tough texture and bitter taste, resulting in significant resource waste (Kwak et al. 2025).

Previous studies have primarily examined the nutritional composition of UPC, whereas APC have received limited attention beyond their basic nutrient profile. Although the APC, including stems and leaves, are known to contain higher levels of vitamin C and essential minerals and have been historically utilized in diverse culinary practices and traditional medicinal applications, their functional and pharmacological properties remain largely unexplored (Kesbiç 2023). To date, there is a lack of systematic investigation into the bioactive potential of APC, as well as anti‐inflammatory activities, which could support their utilization as a valuable resource rather than waste. Therefore, this study aims to fill this gap by comprehensively evaluating the functional and pharmacological potential of celeriac aerial parts.

Phytochemicals are bioactive compounds derived from plants that display a diverse array of physiological functions, including immune‐modulating, anti‐cancer, antioxidant, and anti‐inflammatory properties (Onuh and Pathak 2024). These compounds have garnered increasing attention for their potential in preventing and managing various health conditions (Jang and Lee 2023; Zhang et al. 2019). In particular, celery contains diverse bioactive compounds such as terpenes, coumarins, flavonoids, benzofurans, and phenylpropanoids, all of which have demonstrated inflammation‐reducing, cancer‐fighting, oxidative stress‐reducing, and microbe‐inhibiting properties (Emad et al. 2022; Khairullah et al. 2021). Additionally, phenolic compounds present in celeriac root have shown significant antioxidant and anti‐inflammatory activities (Lee et al. 2021; Popova et al. 2014).

Research has demonstrated that extracts from celery stems and leaves inhibit nitric oxide (NO) production (Lau et al. 2021), suppress cyclooxygenase‐2 (COX‐2) expression (Ramadhan 2020), and reduce the generation of cytokines that promote inflammation (Shin et al. 2019). Moreover, celery contains phytochemicals such as apigenin (APG), apiin, and sedanolide, which have exhibited anti‐inflammatory effects (Che et al. 2021; Lau et al. 2021; Mencherini et al. 2007). Inflammation is a crucial initial response to external pathogens, infections, and physical stimuli, playing an essential role in tissue repair and the maintenance of equilibrium within the body (Medzhitov 2008; Ruiz‐Alcaraz et al. 2024). By modulating the production of inflammation‐inducing cytokines (Hamidzadeh et al. 2017; Rankin 2004), these phytochemicals offer potential therapeutic avenues for preventing inflammation‐related diseases (Chun et al. 2019).

Given the growing interest in plant‐derived bioactive compounds and the need for sustainable utilization of agricultural resources, this study aimed to comprehensively profile the phytochemical composition of both APC and UPC across the entire celeriac plant. Additionally, we sought to evaluate the anti‐inflammatory properties of APC and UPC, with a particular focus on their effects in RAW 264.7 macrophages. Furthermore, the study explored the health‐promoting potential of the identified phytochemicals, emphasizing their possible applications in functional food and pharmaceutical development. This research underscores the potential to repurpose APC into valuable functional food ingredients and pharmaceutical materials.

## 2 Materials and Methods

### 2.1 Chemicals Reagents

Chlorogenic acid (≥ 95%, Cat. No. C3878), 4‐coumaroyl quinic acid (≥ 95%, Cat. No. 935565), 5‐feruloylquinic acid (Phyproof, Cat. No. PHL10013), cryptochlorogenic acid (Phyproof, Cat. No. PHL 80393), ferulic acid (United States Pharmacopeia Reference Standard, Cat. No. 1270311), scopoletin (≥ 99%, Cat. No. S2500), imperatorin (Phyproof, Cat. No. PHL89715), psoralen (≥ 99%, Cat. No. P8399), bergapten (analytical standard, Cat No. 69664), Kaempferol 3‐sambubioside (Phyproof, Cat. No. PHL83517), lipopolysaccharide (LPS, Cat. No. SMB00610), sodium dodecyl sulfate (SDS, Cat. No. 11667289001), and L‐N^6^‐(1‐Iminoethyl) lysine dihydrochloride (L‐NIL, Cat. No. I8021) were purchased from Sigma‐Aldrich (St. Louis, MO, United States). Apiin (99.88%, Cat. No. orb1301262), APG (98.89%, Cat. No. orb1306215), sedanolide (97.14%, Cat. No. orb1301513), and senkyunolide A (≥ 98%, Cat. No. orb1940980) were obtained from Biorbyt (Cambridge, United Kingdom). RAW264.7 macrophages (Cat. No. BS‐4015R), phosphate‐buffered saline (PBS, Cat. No. 10010023), Dulbecco's modified Eagle's medium (DMEM, Cat. No. 11965118), antibiotic‐antimycotic solution (Cat. No. 15240062), and fetal bovine serum (FBS, Cat. No. A5670701) were supplied by Gibco (Thermo Fisher Scientific, Waltham, MA, United States). Water soluble tetrazolium salt 1 (WST‐1, Cat. No. EZ‐3000) was purchased from DoGen Bio (Seoul, Korea).

### 2.2 Sample Preparation

Celeriac was harvested in November 2023 from Haenam County, Jeollanam‐do, South Korea (34°36′30.43′′N, 126°31′53.14′′E). The plants were separated into aerial parts (petioles and leaves) and underground parts (tubers). They were thoroughly washed prior to processing. All samples were stored at −70°C in a deep freezer for a period of 24 h, followed by freeze‐drying for 72 h via a freeze dryer. The freeze‐dried samples were then pulverized into powder form for phytochemical qualitative and quantitative analyses.

To evaluate the anti‐inflammatory efficacy of the samples, 100 g of freeze‐dried APC or UPC powder was mixed with 1 L of 80% (v/v) ethanol. Initial mixing was performed using a vortex mixer to ensure uniform dispersion of the powder in the solvent. The resulting mixture was then transferred to a thermostatically controlled water bath and subjected to extraction at 50°C for 12 h under static conditions, without continuous stirring or agitation. After extraction, the mixture was filtered through Whatman No. 2 filter paper (Whatman Inc., Maidstone, United Kingdom) to remove insoluble residues. This extraction procedure was repeated twice more under identical conditions to maximize the yield of extractable compounds. The three filtrates were combined and concentrated under reduced pressure using a rotary vacuum evaporator (Eyela N‐1000, Tokyo Rikakikai Co., Tokyo, Japan) at 50°C. The concentrated extracts were subsequently lyophilized for 72 h using a freeze‐dryer. The final dried extracts were stored at −70°C in airtight containers until further analysis.

### 2.3 Phytochemical Analysis of APC and UPC

#### 2.3.1 Identification of APC and UPC Phytochemicals

Phytochemical identification was conducted following the methodology outlined by Lee et al. (2024). The freeze‐dried and pulverized APC and UPC samples (0.1 g each) were extracted with 0.1 mL of 80% aqueous methanol, and 1 μL of the resulting extract was injected into the UHPLC‐HRMS system (Thermo Fisher Scientific Inc., California, MA, United States) equipped with a Cortecs T3 column (2.1 mm × 150 mm × 1.6 μm particle size; 120 Å pore size, Waters Co., Milford, MA, United States). The column temperature was maintained at 45°C, with a flow rate of 0.25 mL/min. The sample injection volume was standardized to 1 μL. The mobile phase consisted of two components: (A) 0.1% formic acid in LC/MS‐grade water and (B) 0.1% formic acid in LC/MS‐grade acetonitrile. The gradient elution sequence for the mobile phase was as follows: 1%–3% B from 0 to 5 min, 3%–15% B from 5 to 15 min, 15%–100% B from 15 to 50 min, 100% B from 50 to 55 min, 100%–5% B from 55 to 56 min, and 5% B from 56 to 60 min.

Compound identification was conducted using a Q‐Exactive Plus Orbitap mass spectrometer (Thermo Fisher Scientific), operating in both positive and negative electrospray ionization (ESI) modes. The analysis was performed in data‐dependent MS/MS mode, with a scan range of *m*/*z* 100–1200. Data‐dependent MS/MS acquisition was performed in Top 3 mode, with stepped normalized collision energies (NCEs) of 10, 30, and 50 eV applied using higher‐energy collisional dissociation (HCD). The heated‐ESI (H‐ESI) source parameters were set as follows: capillary voltages of +3800 V (positive mode) and −3000 V (negative mode); ion transfer tube and capillary temperatures at 350°C; sheath gas flow rate of 50 arbitrary units (arb), auxiliary gas flow rate of 10 arb, and sweep gas flow rate of 1 arb.

#### 2.3.2 Quantification of APC and UPC Phytochemicals

Phytochemical quantification was performed based on the methodology outlined by Lee et al. (2024). Freeze‐dried APC and UPC samples (0.1 g) were extracted with 1 mL of 80% aqueous methanol by sonication for 30 min. The extracts were centrifuged, and the supernatant was collected for analysis. A 1 μL aliquot of the resulting extract was injected into a Vanquish Flex UHPLC system (Thermo Fisher Scientific Inc., Waltham, MA, United States) equipped with a CORTECS C18 column (2.1 mm × 150 mm, 1.6 μm particle size; 120 Å pore size, Waters Co.). The column was maintained at 45°C, with a 1 μL injection volume and a flow rate of 0.3 mL/min.

Two mobile phases were used for the gradient elution: Mobile Phase A consisted of 0.1% formic acid in LC/MS‐grade water, while Mobile Phase B contained 0.1% formic acid in LC/MS‐grade methanol. The gradient program included: 5% B from 0 to 0.5 min, increasing to 25% B from 0.5 to 1 min, followed by 100% B from 1 to 10 min. The gradient was held at 100% B from 10 to 10.5 min, reduced to 5% B from 10.5 to 11 min, and maintained at 5% B from 11 to 15 min.

Quantitative analysis was carried out using a Thermo TSQ Altis triple quadrupole mass spectrometer, equipped with a H‐ESI source in selected reaction monitoring (SRM) mode with polarity switching. Table S1 presents the optimized SRM parameters for the 14 quantified phytochemical compounds, including adduct type, precursor ion, quantifier ion, qualifier ions, and their corresponding collision energy values. The H‐ESI source was configured with the following parameters: sheath gas flow rate at 50 arb, auxiliary gas flow rate at 10 arb, and sweep gas flow rate at 1 arb. The capillary voltage was set to +3500 V for positive ionization mode and −2500 V for negative ionization mode. Both the ion transfer tube and capillary were maintained at 350°C. Data collection and analysis were performed with Trace Finder 4.1 software (Thermo Fisher Scientific Inc., Waltham, MA, United States). Method validation was conducted to evaluate the reliability and robustness of the analytical method. The validation parameters included retention time (RT), linear range, coefficient of determination (*R* ^2^), limit of detection (LOD), limit of quantification (LOQ), accuracy, and precision (Table S2). Linearity and *R* ^2^ values were determined using calibration curves generated from authentic standards (Table S2 and Figure S1). Accuracy and precision were assessed by spiking 100 ng/mL of each standard compound into 0.1 g of a blank matrix, followed by extraction with 80% aqueous methanol and analysis under the same conditions as the test samples. LC–MS/MS chromatograms of the identified phytochemicals and their corresponding reference standards are provided in Figure S2. These chromatograms visually demonstrate the alignment of RTs and peak patterns between sample analytes and authentic standards.

#### 2.3.3 Compound Identification and Data Analysis

For compound annotation, raw chromatography data were processed using Scaffold Elements 2.21 with automated peak detection. Concurrently, mass spectrum deconvolution was conducted using established spectral libraries, including the National Institute of Standards and Technology (NIST), MassBank of North America (MoNA), and Human Metabolome Database (HMDB). A heatmap was generated using MetaboAnalyst (https://www.metaboanalyst.ca/) based on relative peak intensities of phytochemicals identified in different parts of celeriac.

### 2.4 In Vitro Anti‐Inflammatory Effects of APC and UPC Extracts

#### 2.4.1 Cell Culture

RAW 264.7 cells were procured from the American Type Culture Collection (ATCC) (Manassas, VA, United States) and cultured in DMEM supplemented with 10% FBS and 1% antibiotic‐antimycotic solution. Cells were maintained at 37°C in a humidified incubator with 5% CO_2_. When cell confluency reached approximately 80%, they were washed with PBS, detached using a cell scraper, and subsequently collected via centrifugation at 1000 rpm for a duration of 3 min. The culture medium was replenished at 48‐h intervals.

#### 2.4.2 Assessment of Cell Viability

Cell viability was evaluated using a WST‐1 assay. RAW 264.7 cells were seeded in a 96‐well plate at a density of 3 × 10^4^ cells per well and treated with various concentrations (0.01–1 mg/mL) of APCE and UPCE. A 1 mg/mL SDS solution served as a positive control for the induction of cell death. The cells were incubated at 37°C with 5% CO_2_ for 24 h. After incubation, 10 μL of WST‐1 reagent was added to each well, followed by a 1‐h incubation at 37°C. Absorbance was measured at 450 nm using a microplate reader (Thermo Fisher Scientific, Waltham, MA, United States).

#### 2.4.3 Measurement of NO Production

RAW 264.7 cells were plated in 6‐well plates at a density of 8 × 10^5^ cells per well and pretreated with APCE (0.05, 0.1, 0.5 mg/mL), UPCE (0.01, 0.05, 0.1 mg/mL), APG (50 μM), or L‐NIL (20 mM) for 1 h. Following pretreatment, the cells in each well were treated with 1 μg/mL of LPS. Subsequently, the cells were incubated at 37°C for 24 h. NO levels were quantified using the Griess Reagent System (Promega, Madison, WI, United States). Absorbance was measured at 540 nm using a SpectraMax ABS microplate reader (Molecular Devices, San Jose, CA, United States). Nitrite concentrations (μM) were determined using a sodium nitrite standard curve with the nitrite solution provided in the Griess Reagent System Kit. APG and L‐NIL served as standard controls.

#### 2.4.4 Quantitative Real‐Time PCR

RAW 264.7 cells were plated in 6‐well plates at a density of 8 × 10^5^ cells per well and treated with APCE (0.05, 0.1, 0.5 mg/mL) or UPCE (0.01, 0.05, 0.1 mg/mL) for 1 h prior to further experimentation. Subsequently, LPS (1 μg/mL) was added, and the cells were incubated at 37°C for 24 h. APG was used as a standard control in the experiment.

To assess inflammatory gene expression, cells were harvested, pelleted, and stored at −80°C. RNA extraction was performed using the RNeasy Mini Kit (Qiagen, Hilden, Germany), and its purity was evaluated by measuring absorbance at 260 and 280 nm. Only RNA samples with an OD260/280 ratio ≥ 1.8 were employed for complementary DNA (cDNA) synthesis and quantitative polymerase chain reaction (qPCR) analysis. Reverse transcription of RNA into cDNA was performed using a TOPscript cDNA Synthesis Kit (Enzynomics, Daejeon, Korea). The synthesized cDNA was amplified via qPCR using TOPreal SYBR Green qPCR Premix (Enzynomics, Daejeon, Korea) and specific primers following the manufacturer's instructions. Quantitative qPCR was performed using a MIC instrument (BMS, Australia). Primer sequences utilized in qPCR are documented in Table 1. The qPCR results were analyzed using Ct (threshold cycle) values, with glyceraldehyde‐3‐phosphate dehydrogenase (GAPDH) serving as the reference gene. The target gene expression levels in the experimental groups were assessed in relation to the control group through the calculation of the 2^−ΔΔCt^ method.

**TABLE 1:** List of primers used for quantitative real‐time PCR.

Gene	Direction	Primer sequence
GAPDH	Forward	CAA GGC TGT GGG CAA GGT
	Reverse	GGA AGG CCA TGC CAG TGA
COX‐2	Forward	GAA GAT TCC CTC CGG TGT TT
	Reverse	CCC TTC TCA CTG GCT TAT GTA G
TNF‐α	Forward	TAT GGC TCA GGG TCC AAC TC
	Reverse	CTC CCT TTG CAG AAC TCA GG
IL‐6	Forward	GGT GAC AAC CAC GGC CTT CCC
	Reverse	AAG CCT CCG ACT TGT GAA GTG GT
IL‐1β	Forward	TTG ACG GAC CCC AAA AGA TG
	Reverse	AGA AGG TGC TCA TGT CCT CA
iNOS	Forward	CAG CAC AGG AAA TGT TTC AGC
	Reverse	TAG CCA GCG TAC CGG ATG A

Abbreviations: COX‐2, cyclooxygenase‐2; GAPDH, glyceraldehyde 3‐phosphate dehydrogenase; iNOS, inducible nitric oxide synthase; IL‐1β, interleukin‐1 beta; IL‐6, interleukin‐6; PCR, polymerase chain reaction; TNF‐α, tumor necrosis factor‐alpha.

#### 2.4.5 Enzyme‐Linked Immunosorbent Assay (ELISA)

RAW 264.7 cells were plated in 6‐well plates at a density of 8 × 10^5^ cells per well. Each well was pretreated with APCE (0.05, 0.1, 0.5 mg/mL), UPCE (0.01, 0.05, 0.1 mg/mL), or APG for 1 h, followed by the addition of LPS (1 μg/mL) to each well. The cells were then incubated at 37°C for 24 h. After incubation, the supernatants were harvested from each well and kept at −80°C. For ELISA assays, the supernatants were thawed and examined using a prostaglandin E2 ELISA kit (Cayman Chemical Co., Ann Arbor, United States) following the manufacturer's instructions. The measurements of optical density were conducted at 405 nm using a SpectraMax ABS microplate reader.

### 2.5 Statistical Analysis

Results are expressed as average values with their standard deviations (SDs). Group differences were assessed through analysis of variance (ANOVA), followed by Duncan's multiple range test for post hoc comparisons. For pairwise comparisons, independent samples *t*‐tests were conducted. A *p*‐value of less than 0.05 was considered statistically significant.

## 3 Results

### 3.1 Phytochemical Compositions

#### 3.1.1 Identification of Phytochemicals

Phytochemical compounds present in APC and UPC were identified using a UHPLC‐HRMS system. The analysis was performed utilizing both positive and negative ionization modes (Figures S3 and S4). As summarized in Table 2, a total of 31 distinct compounds were detected in APC and UPC. These included 9 hydroxycinnamic acids, 2 hydroxycoumarins, 1 flavonol, 8 flavones, 8 furanocoumarins, and 3 phthalides.

**TABLE 2:** Phytochemicals identified from aerial and underground parts of celeriac.

	Compounds	RT (min)	Molecular formula	Molecular weight	Precursor ions (*m*/*z*)	Mass error (ppm)	Adduct	MS/MS fragment ions (*m*/*z*)	Average of peak area × 10^6^ (*n* = 3)	
									APC	UPC
Hydroxycinnamic acids										
1	Caffeic acid hexoside	10.184	C_15_H_18_O_9_	342.0950	341.0879	0.24	[M−H]^−^	135.0441, 179.0342, 341.0879, 134.0360, 283.4684	14.047	24.720
2	Chlorogenic acid	10.856	C_16_H_18_O_9_	354.0949	353.0876	0.56	[M−H]—	191.0556, 85.0282, 353.0897, 93.0333, 135.0436	2866.117	730.685
3	Caffeic acid	11.184	C_9_H_8_O_4_	180.0415	179.0342	−2.47	[M−H]^−^	135.0442, 179.0341, 134.0365, 90.9968, 134.9871	46.076	11.813
4	Cryptochlorogenic acid	11.410	C_16_H_18_O_9_	354.0952	353.0879	0.22	[M−H]‐	191.0557, 135.0442, 173.0448, 179.0343, 93.0334	91.351	15.707
5	1‐Caffeoylquinic acid	13.254	C_16_H_18_O_9_	354.0951	353.0878	−0.01	[M−H]^−^	191.0556, 85.0282, 353.0873, 93.0331, 127.0384	259.261	3.153—
6	4‐Coumaroylquinic acid	14.027	C_16_H_18_O_8_	338.1003	337.0930	0.27	[M−H]^−^	191.0557, 93.0333, 119.0490, 163.0391, 87.0075	267.648	17.344
7	5‐Feruloylquinic acid	15.923	C_17_H_20_O_9_	368.1106	367.1034	0.24	[M−H]^−^	191.0555, 93.0333, 134.0363, 87.0074, 193.0498	218.238	45.147
8	1‐Coumaroylquinic acid	16.199	C_16_H_18_O_8_	338.1003	337.0930	0.32	[M−H]^−^	191.0558, 85.0281, 93.0333, 127.0389, 337.0939	402.335	3.562
9	Ferulic acid	17.556	C_10_H_10_O_4_	194.0572	193.0500	−2.46	[M−H]^−^	134.0364, 178.0266, 193.0502, 137.0236, 90.9322	8.822	10.222
Hydroxycoumarins										
1	Esculin	8.687	C_15_H_16_O_9_	340.0794	339.0721	−0.13	[M−H]^−^	177.0186, 339.0720, 333.5693, 280.9667, 133.0287	8.822	10.222
2	Scopoletin	17.240	C_10_H_8_O_4_	192.0415	191.0343	−2.73	[M−H]^−^	176.0107, 148.0156, 191.0344, 104.0255, 120.0204	20.935	63.752
Flavonol										
1	Kaempferol 3‐sambubioside	20.152	C_26_H_28_O_15_	580.1424	579.1352	−0.66	[M−H]^−^	285.0411, 284.0330, 579.1357, 284.0029, 151.0026	553.471	6.545
Flavones										
1	Cynaroside	20.177	C_21_H_20_O_11_	448.1005	447.0933	−0.05	[M−H]^−^	285.0409, 284.0332, 447.0935, 151.0027, 284.0644	43.417	—
2	Apiin	21.335	C_26_H_28_O_14_	564.1474	563.1402	−0.86	[M−H]^−^	269.0459, 563.1392, 268.0389, 492.7634, 145.6883	915.687	225.395
3	Diosmetin‐7‐O‐arabinoglucoside	21.725	C_27_H_30_O_15_	594.1588	593.1515	0.47	[M−H]^−^	299.0565, 284.0331, 593.1520, 255.0301, 283.0254	143.148	43.238
4	Luteolin 7‐O‐(6″‐malonylglucoside)	21.865	C_24_H_22_O_14_	534.1010	533.0938	0.14	[M−H]^−^	285.0411, 284.0332, 489.1055, 91.9568, 256.0385	145.834	0.594
5	Diosmetin‐7‐O‐glucoarabinoside	21.970	C_27_H_30_O_15_	594.1586	593.1514	0.26	[M−H]^−^	284.0329, 299.0564, 593.1522, 91.9568, 558.1734	31.582	58.649
6	6″‐Malonylapiin	22.763	C_29_H_30_O_17_	650.1482	649.1409	−0.14	[M−H]^−^	269.0459, 605.1524, 268.0376, 563.1451, 117.0331	830.980	742.131
7	Diosmetin	23.393	C_16_H_12_O_6_	300.0633	299.0563	0.53	[M−H]^−^	284.0332, 299.0566, 256.0381, 151.0028, 183.1020	19.802	1.259
8	Apigenin	25.995	C_15_H_10_O_5_	270.0530	269.0458	0.74	[M−H]^−^	269.0458, 117.0334, 151.0029, 149.023, 107.0126	39.346	2.302
Furanocoumarins										
1	Psoralen	25.029	C_11_H_6_O_3_	186.0316	187.0388	−0.77	[M + H]^+^	187.0393, 131.0495, 115.0547, 143.0494, 188.0428	113.3	91.466
2	Fraxinol methyl ether	25.402	C_12_H_12_O_5_	236.0684	237.0756	−0.82	[M + H]^+^	237.0760, 222.0525, 207.0291, 193.0499, 191.0341	45.803	0.569
3	Isobergaptene	26.017	C_12_H_8_O_4_	216.0419	217.0492	−1.54	[M + H]^+^	217.0500, 202.0265, 161.0598, 174.0314, 189.0550	2604.441	3734.746
4	Citropten	26.937	C_11_H_10_O_4_	206.0577	207.0650	−0.89	[M + H]^+^	207.0656, 192.0420, 163.0756, 151.0755, 164.0471	436.407	26.175
5	Bergapten	27.537	C_12_H_8_O_4_	216.0419	217.0492	−1.55	[M + H]^+^	217.0499, 202.0266, 217.0173, 189.0188, 174.0314	2179.144	2187.017
6	Isopimpinellin	27.736	C_13_H_10_O_5_	246.0528	247.0596	−2.01	[M + H]^+^	247.0604, 217.0135, 232.0369, 189.0186, 248.064	3838.332	3334.198
7	Peucenin	30.004	C_15_H_16_O_4_	260.1047	261.1120	−0.68	[M + H]^+^	205.0500, 261.1125, 190.0264, 162.0315, 121.0651	8.344	49.048
8	Imperatorin	33.095	C_16_H_14_O_4_	270.0889	271.0961	−1.32	[M + H]^+^	203.0342, 69.0706, 147.0443, 175.0392, 131.0495	137.005	122.698
Phthalides										
1	Senkyunolide A	31.174	C_12_H_16_O_2_	192.1147	193.1220	−1.58	[M + H]^+^	137.0600, 147.1170, 91.0548, 93.0704, 175.1120	7929.883	1508.818
2	Sedanolide	33.380	C_12_H_18_O_2_	194.1305	195.1377	−1.12	[M + H]^+^	79.0549, 149.1328, 125.0600, 177.1277, 81.0706	1997.585	586.373
3	Ligustilide	33.562	C_12_H_14_O_2_	190.0993	191.1065	−0.59	[M + H]^+^	191.1071, 173.0965, 145.1016, 91.0548, 117.0705	120.406	183.526

*Note:* Different compounds were detected in APC and UPC by performing UHPLC‐HRMS analysis.

Abbreviations: APC, aerial parts of celeriac; RT, retention time; UHPLC‐HRMS, ultra‐high performance liquid chromatography–high‐resolution mass spectrometry; UPC, underground parts of celeriac.

**FIGURE 1:**
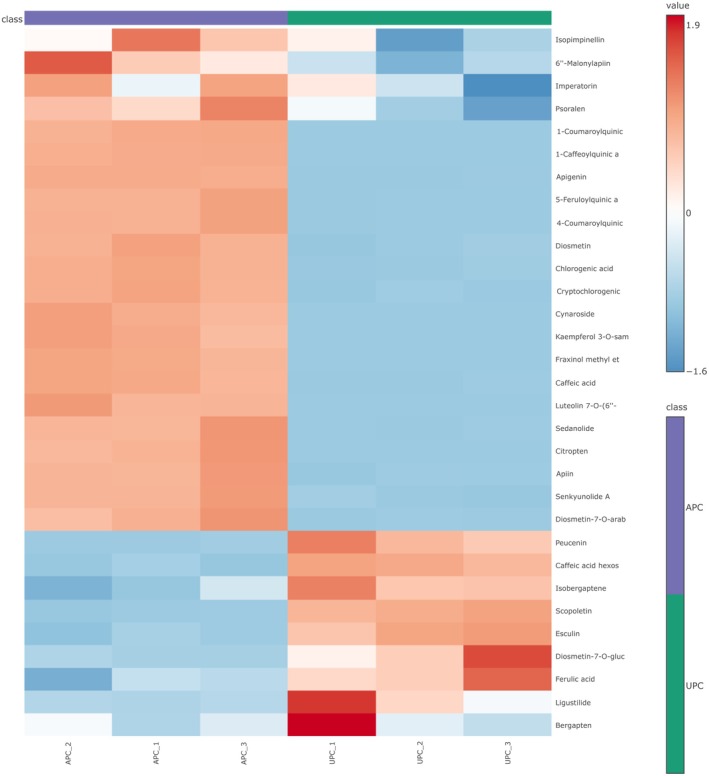
Heat map showing relative abundance of different compounds detected in aerial and underground parts of *celeriac*. This heat map was constructed using the web‐based MetaboAnalyst platform. APC, aerial parts of celeriac; UPC, underground parts of celeriac.

Figure 1 presents a heatmap comparing relative peak intensities of the 31 compounds identified in APC and UPC. The heatmap used a color gradient to represent relative peak intensities, with red indicating higher peak values and blue representing lower peak values. Most compounds displayed higher peak values in APC, as reflected by a predominant red color, whereas UPC exhibited comparatively lower peak values. Notably, a total of 9 compounds (peucenin, caffeic acid hexoside, isobergaptene, scopoletin, esculin, diosmetin‐7‐O‐glucoarabinoside, ferulic acid, ligustilide, and bergapten) exhibited higher peak values (red) in UPC compared to APC. These findings revealed significant differences in compound profiles between APC and UPC, with certain compounds being notably more abundant in APC. This highlights the distinct chemical compositions of these two plant parts.

#### 3.1.2 Quantification of Phytochemicals

**FIGURE 2:**
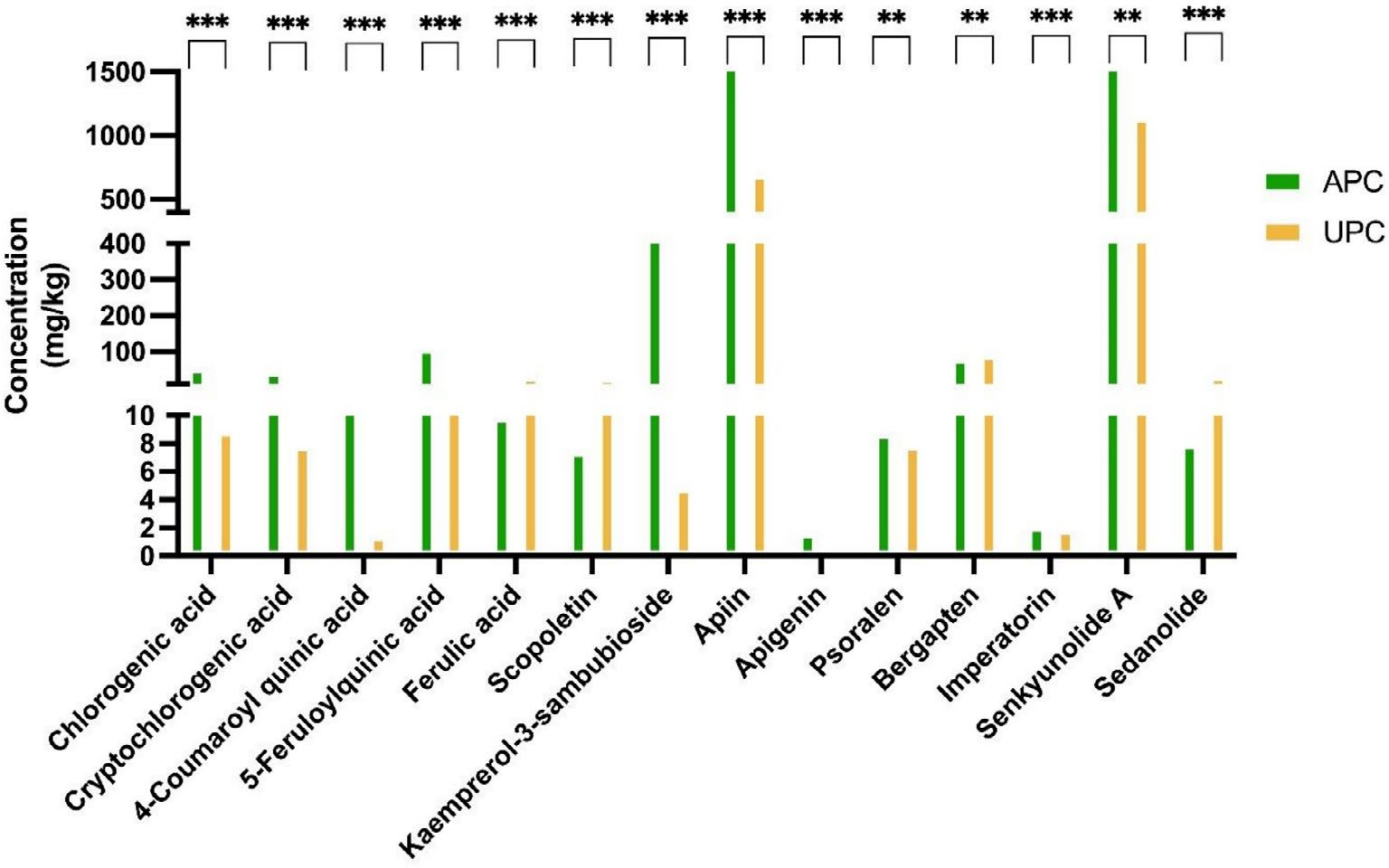
Phytochemical compositions of aerial and underground parts of celeriac. All data are presented as mean ± standard deviation (*n* = 3). ***p < 0.001; **p < 0.01 compared with each compound. APC, aerial parts of celeriac; UPC, underground parts of celeriac.

The primary phytochemicals in APC and UPC were analyzed to identify compounds contributing to their anti‐inflammatory effects and health benefits. A total of 14 compounds were quantified (Table 3, Figures 2 and S5). Among hydroxycinnamic acids, chlorogenic acid, cryptochlorogenic acid, 4‐coumaroylquinic acid, and 5‐feruloylquinic acid exhibited significantly higher concentrations in APC than in UPC. In contrast, ferulic acid showed a significantly higher concentration in UPC. In the category of hydroxycoumarin, scopoletin demonstrated significantly higher levels in UPC than in APC. For flavonol, kaempferol 3‐sambubioside was significantly more abundant in APC than in UPC. Similarly, in the flavones category, apiin and APG also displayed significantly higher concentrations in APC. Within furanocoumarins, psoralen and imperatorin were present at higher concentrations in APC, whereas bergapten exhibited significantly higher levels in UPC. In the category of phthalides, sedanolide was more abundant in UPC, whereas senkyunolide A was significantly more abundant in APC. These quantitative findings are in agreement with the distribution patterns illustrated in the heatmap shown in Figure S5.

**TABLE 3:** Phytochemical compositions of aerial and underground parts of celeriac.

	APC	UPC	*T*
*Hydroxycinnamic acids*			
Chlorogenic acid	53.37 ± 1.83	8.88 ± 0.17	−41.774***
Cryptochlorogenic acid	45.06 ± 0.56	7.83 ± 0.13	−111.237***
4‐Coumaroyl quinic acid	15.58 ± 0.35	1.41 ± 0.21	−69.202***
5‐Feruloylquinic acid	109.30 ± 0.49	21.98 ± 0.24	−273.354***
Ferulic acid	9.86 ± 0.71	32.03 ± 2.10	17.295***
*Hydroxycoumarin*			
Scopoletin	7.41 ± 0.23	28.90 ± 0.28	100.032***
*Flavonol*			
Kaempferol 3‐sambubioside	418.65 ± 2.75	4.85 ± 0.14	−259.653***
*Flavones*			
Apiin	3232.27 ± 112.27	697.06 ± 7.90	−39.012***
Apigenin	1.63 ± 0.17	0.10 ± 0.00	−155.214***
*Furanocoumarins*			
Psoralen	8.68 ± 0.16	7.87 ± 0.98	−7.311**
Bergapten	80.66 ± 1.33	91.05 ± 1.19	10.033**
Imperatorin	2.10 ± 0.30	1.86 ± 0.10	−12.908***
*Phthalides*			
Senkyunolide A	15,194.26 ± 2021.96	1145.04 ± 12.50	−12.035**
Sedanolide	7.96 ± 0.16	33.00 ± 1.65	26.083***

*Note:* All data are expressed as mean ± standard deviation (*n* = 3), with values presented in mg/kg.

Abbreviations: APC, aerial parts of celeriac; UPC, underground parts of celeriac.

** *p* < 0.01 compared with each compound.

*** *p* < 0.001.

### 3.2 In Vitro Evaluation of the Anti‐Inflammatory Properties of APCE and UPCE

#### 3.2.1 Effects of APCE and UPCE on Cell Viability

**FIGURE 3:**
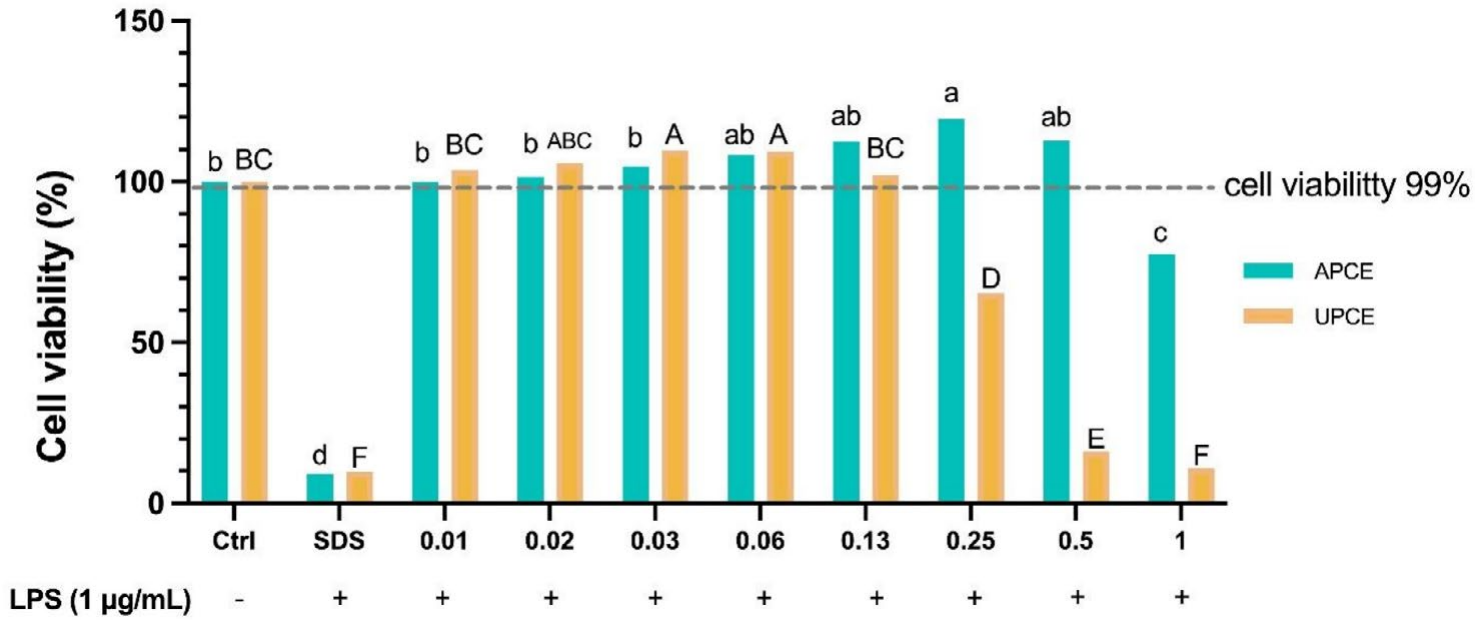
Effects of APCE and UPCE on the cell viability of RAW 264.7 cells. Cells were treated with increasing concentrations (0.01–1 mg/mL) of extracts for 24 h. Cell viability was measured using a WST‐1 cell viability assay kit. Results are expressed as cell viability (% of control). All data are presented as mean ± standard deviation (*n* = 3). ^(a–d,A–F)^Mean values with different letters are significantly different (*p* < 0.05) among groups as determined by Duncan's multiple range test. APCE, aerial parts of celeriac extracts; LPS, lipopolysaccharide; SDS, sodium dodecyl sulfate; UPCE, underground parts of celeriac extracts.

APCE and UPCE did not exhibit cytotoxic effects within concentration ranges of 0.01–0.5 mg/mL for APCE and 0.01–0.13 mg/mL for UPCE, resulting in cell viability exceeding 99% relative to the control group (Figure 3 and Table S3). Based on these findings, it was established that APCE did not affect cell viability at concentrations below 0.5 mg/mL, while UPCE did not show any effect at concentrations below 0.13 mg/mL. Therefore, for further experiments, concentrations of 0.05, 0.1, and 0.5 mg/mL for APCE and 0.01, 0.05, and 0.1 mg/mL for UPCE were chosen due to their non‐cytotoxic nature.

#### 3.2.2 Effects of APCE and UPCE on NO Production and iNOS Expression

**FIGURE 4:**
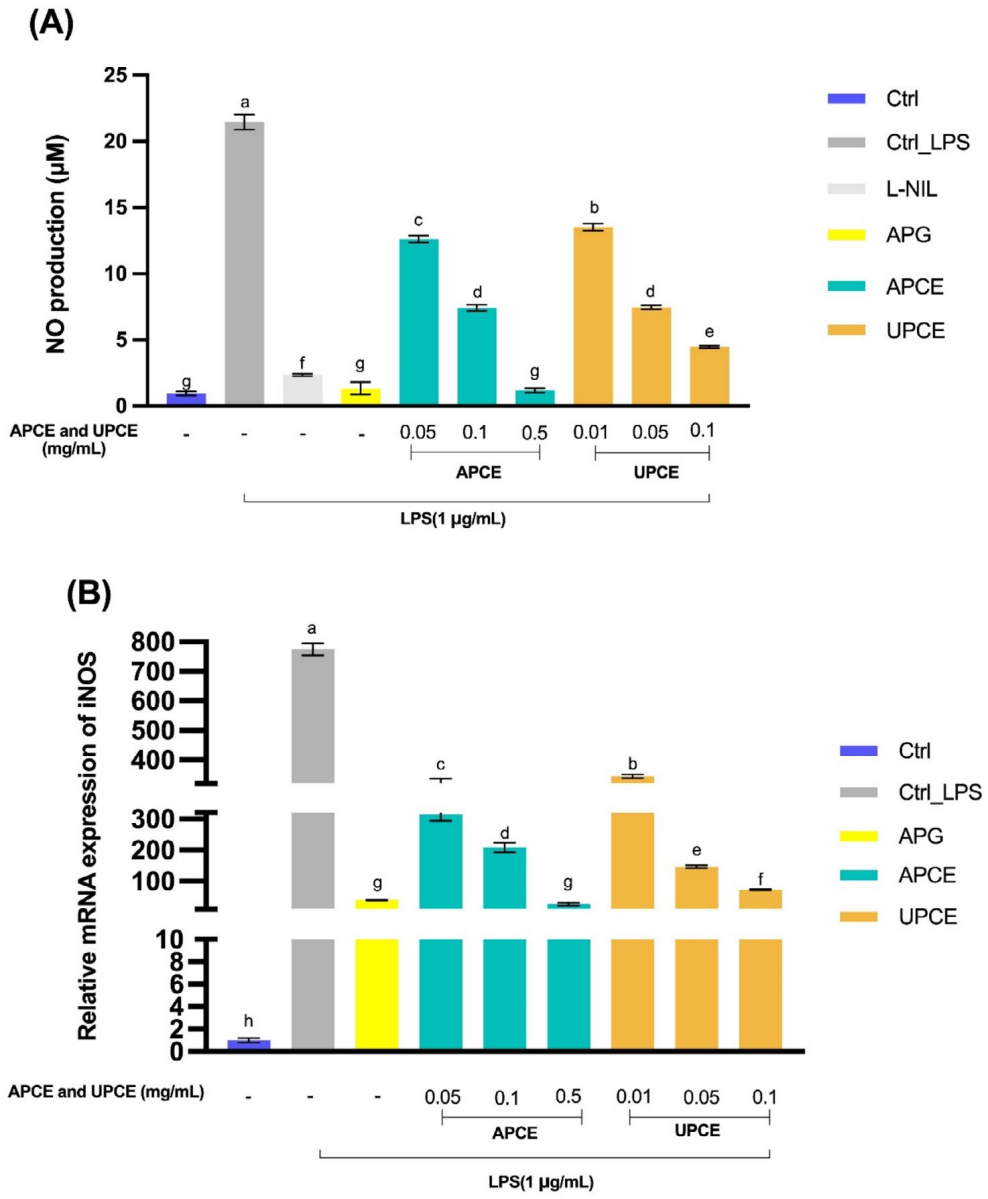
Effects of APCE and UPCE on NO production and *iNOS* mRNA expression in LPS‐stimulated RAW 264.7 cells. (A) Effects of APCE and UPCE on NO production in RAW 264.7 cells. After treating cells with LPS, APG (50 μM), L‐NIL (20 mM), APCE (0.05, 0.1, and 0.5 mg/mL), or UPCE (0.01, 0.05, and 50 mg/mL), NO production was measured by Griess assays. (B) Effects of APCE and UPCE on *iNOS* mRNA expression in RAW 264.7 cells. After culturing cells with LPS, APG (50 μM), APCE (0.05, 0.1, and 0.5 mg/mL), and UPCE (0.01, 0.05, and 0.1 mg/mL), mRNA expression of *iNOS* was determined by qRT‐PCR. All data are presented as mean ± standard deviation (*n* = 3). ^(a–h)^Mean values with different letters are significantly different (*p* < 0.05) among groups as determined by Duncan's multiple range test. APCE, aerial parts of celeriac extract; APG, apigenin; iNOS, inducible nitric oxide synthase; L‐NIL, N6‐(1‐iminoethyl)‐L‐lysine hydrochloride; LPS, lipopolysaccharide; NO, nitric oxide; qRT‐PCR, quantitative reverse transcription polymerase chain reaction; UPCE, underground parts of celeriac extract.

The effects of APCE and UPCE on NO production were assessed through the Griess assay. The data presented in Figure 4A indicate an increase in NO production in the LPS‐treated group, reaching 21.45 μM relative to the control group (*p* < 0.05). Nonetheless, a significant reduction was observed in NO production with APCE and UPCE across all measured concentrations (*p* < 0.05). L‐NIL and APG, as positive controls, also significantly decreased NO levels (*p* < 0.05). Notably, APCE at 0.5 mg/mL exhibited the greatest inhibitory effect, reducing the NO level to 1.20 μM, comparable to the NO level in the APG‐treated group. When NO production levels were compared between groups treated with APCE and UPCE at the same concentrations (0.05 and 0.1 mg/mL), the group treated with UPCE showed significantly lower NO levels (*p* < 0.05).

Inducible nitric oxide synthase (iNOS) is induced in macrophages in response to inflammatory stimuli and promotes NO production (Anavi and Tirosh 2020). In Figure 4B, iNOS mRNA expression decreased proportionally with concentration in response to APCE or UPCE in contrast to the LPS‐treated group (*p* < 0.05). APG, used as a positive control, demonstrated a statistically meaningful decrease in iNOS expression (*p* < 0.05). In line with the findings on NO production, APCE at 0.5 mg/mL induced the most pronounced decrease in iNOS mRNA expression, reaching a level comparable to that observed in the APG‐treated group. At equivalent concentrations, the UPCE‐treated group exhibited a statistically significant reduction in iNOS mRNA levels, comparable to the APCE‐treated group (*p* < 0.05).

#### 3.2.3 Effects of APCE and UPCE on COX‐2 mRNA Expression and PGE_2_ Production

**FIGURE 5:**
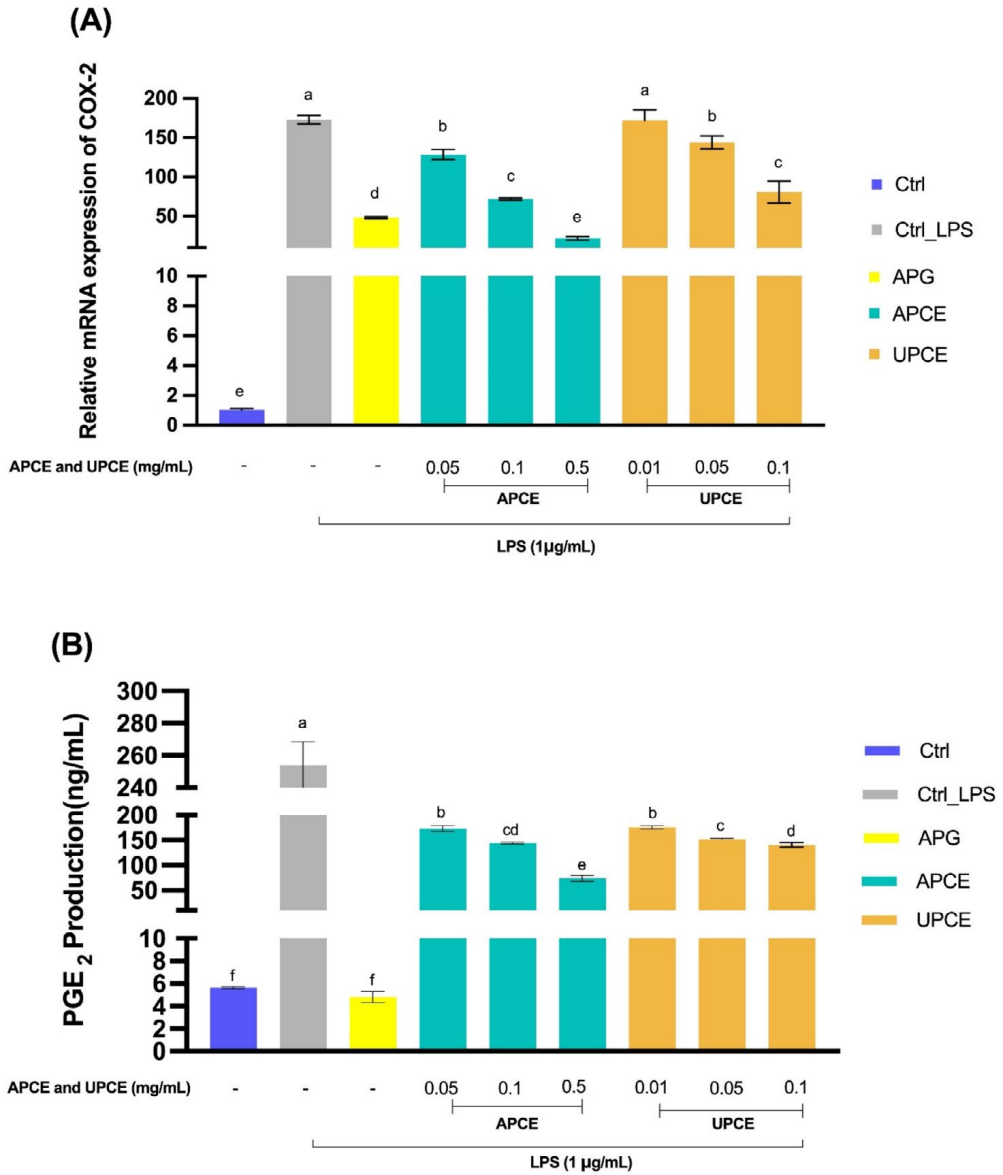
Effects of APCE and UPCE on COX‐2 mRNA expression and PGE₂ production in LPS‐stimulated RAW 264.7 cells. (A) Effects of APCE and UPCE on COX‐2 mRNA expression in RAW 264.7 cells. After treating cells with LPS, APG (50 μM), APCE (0.05, 0.1, and 0.5 mg/mL), or UPCE (0.01, 0.05, and 0.1 mg/mL), mRNA expression levels of COX‐2 were determined by qRT‐PCR. (B) Effects of APCE and UPCE on PGE_2_ production in RAW 264.7 cells. After treating cells with LPS, APG (50 μM), APCE (0.05, 0.1, and 0.5 mg/mL), or UPCE (0.01, 0.05, and 0.1 mg/mL), PGE_2_ production was measured using ELISA. All data are presented as mean ± standard deviation (*n* = 3). ^(a–f)^Mean values with different letters are significantly different (*p* < 0.05) among groups as determined by Duncan's multiple range test. APCE, aerial parts of celeriac extract; APG, apigenin; COX‐2, cyclooxygenase‐2; PGE_2_, prostaglandin E₂; LPS, lipopolysaccharide; qRT‐PCR, quantitative reverse transcription polymerase chain reaction; UPCE, underground parts of celeriac extract.

The enzyme COX‐2 plays a significant role in inflammation by facilitating the synthesis of PGE_2_ through prostaglandin production (Chien et al. 2015). The effects of APCE and UPCE on COX‐2 mRNA expression and PGE_2_ production are shown in Figure 5. The group treated with LPS exhibited an increase in both COX‐2 mRNA expression and PGE_2_ production relative to the control group (*p* < 0.05). APCE treatment resulted in a marked reduction of COX‐2 mRNA expression, showing a concentration‐dependent relationship. Notably, when administered at the highest concentration of 0.5 mg/mL, APCE reduced COX‐2 mRNA expression below levels observed in the APG‐treated group, demonstrating a strong anti‐inflammatory effect of APCE (*p* < 0.05). In the group exposed to UPCE at the lowest concentration (0.01 mg/mL), COX‐2 mRNA expression showed a reduction compared to that in the LPS‐treated group, although such reduction was not statistically significant. However, at doses of 0.05 and 0.1 mg/mL, UPCE notably lowered expression of COX‐2 mRNA levels (*p* < 0.05). When the effects of APCE and UPCE at the same concentrations were compared, APCE resulted in lower COX‐2 mRNA levels, although their differences were not statistically significant (Figure 5A).

APCE and UPCE also exhibited a concentration‐dependent reduction in PGE_2_ production (*p* < 0.05). The most notable reduction in PGE_2_ production was noticed in the APG treated group, bringing levels down to those comparable to the control group. Among the extracts at different concentrations, APCE at 0.5 mg/mL resulted in the greatest reduction of PGE_2_ production. When comparing the two extracts at the same concentrations, UPCE at 0.05 mg/mL led to a more marked decrease in PGE_2_ synthesis than APCE (*p* < 0.05). However, at 0.1 mg/mL, the two extracts showed no significant difference in decreasing PGE_2_ production (Figure 5B).

#### 3.2.4 Effects of APCE and UPCE on mRNA Expression Levels of Pro‐Inflammatory Cytokines

**FIGURE 6:**
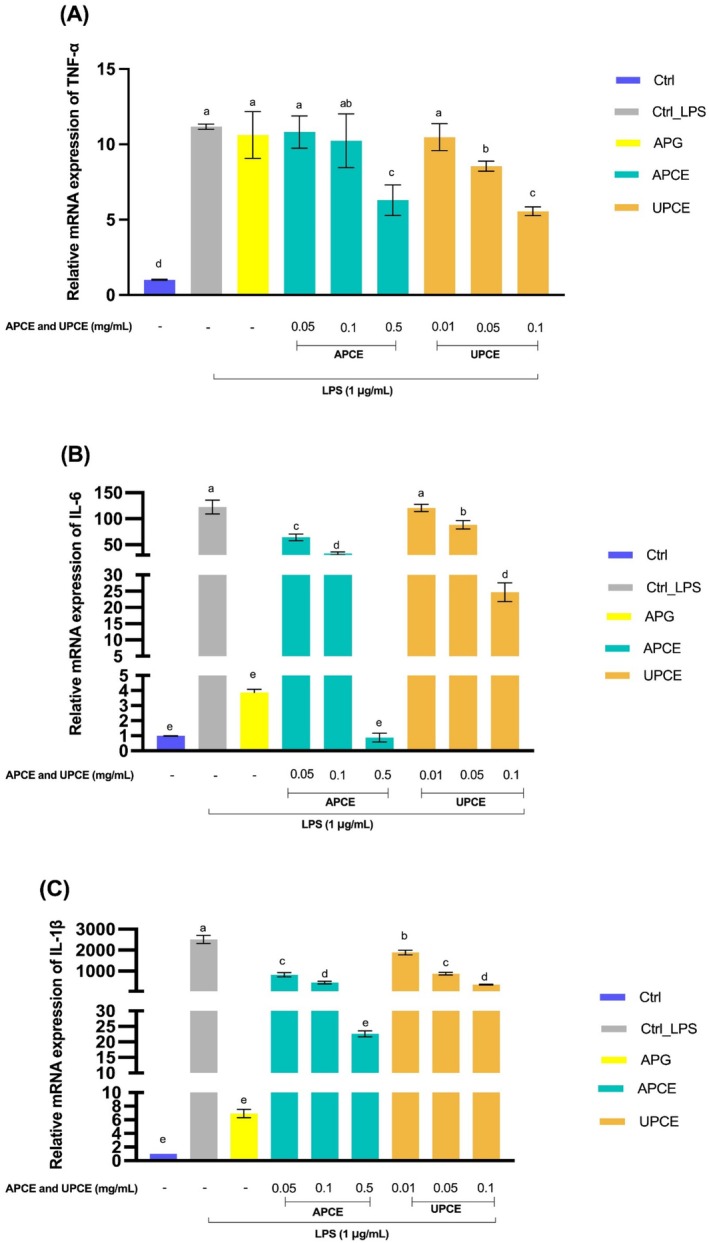
Effects of APCE and UPCE on inflammation‐related cytokines. After treating cells with LPS, APG (50 μM), APCE (0.05, 0.1, and 0.5 mg/mL), or UPCE (0.01, 0.05, and 0.1 mg/mL), mRNA expression levels of TNF‐α (A), IL‐6 (B), and IL‐1β (C) were determined by qRT‐PCR. All data are presented as mean ± standard deviation (*n* = 3). ^(a–e)^Mean values with different letters are significantly different (*p* < 0.05) among groups as determined by Duncan's multiple range test. APCE, aerial parts of celeriac extract; APG, apigenin; IL‐1β, interleukin‐1 beta; IL‐6, interleukin‐6; TNF‐α, tumor necrosis factor‐alpha; LPS, lipopolysaccharide; qRT‐PCR, quantitative reverse transcription polymerase chain reaction; UPCE, underground parts of celeriac extract.

The effects of APC and UPC extracts on mRNA expression levels of pro‐inflammatory cytokines were assessed through qRT‐PCR. The transcription levels of TNF‐α, IL‐6, and IL‐1β are shown in Figure 6. The LPS‐treated group showed significantly elevated inflammatory marker expression compared to the control group (*p* < 0.05). For TNF‐α mRNA expression (Figure 6A), both APCE and UPCE decreased their expression levels proportionally to the concentration levels. APCE (0.1 and 0.5 mg/mL) and UPCE (0.05 and 0.1 mg/mL) concentrations resulted in a statistically meaningful reduction in the expression of TNF‐α mRNA levels (*p* < 0.05). When comparing APCE and UPCE at the same concentrations, UPCE resulted in significantly lower TNF‐α mRNA levels than APCE (*p* < 0.05).

APCE exhibited a concentration‐dependent reduction in IL‐6 mRNA expression levels, as shown in Figure 6B (*p* < 0.05). UPCE at dosages of 0.05 and 0.1 mg/mL also notably decreases IL‐6 mRNA expression (*p* < 0.05). At 0.5 mg/mL, APCE reduced IL‐6 mRNA expression to levels comparable to those in untreated control and APG‐treated groups, indicating its strong anti‐inflammatory effect. When comparing APCE and UPCE at the same concentrations, APCE at 0.05 mg/mL resulted in lower expression levels of IL‐6 mRNA (*p* < 0.05), whereas UPCE at 0.1 mg/mL resulted in lower expression levels of IL‐6 mRNA (*p* < 0.05).

APCE and UPCE both showed a decrease in IL‐1β mRNA expression levels that depended on the concentration, as indicated in Figure 6C (*p* < 0.05). The group that underwent APG treatment demonstrated the lowest level of expression. The 0.5 mg/mL APCE significantly decreased IL‐1β mRNA expression to levels comparable to those observed in the APG group, indicating a strong anti‐inflammatory impact.

## 4 Discussion

This study comprehensively profiled the phytochemical composition of celeriac and explored its health benefits, with a particular focus on its anti‐inflammatory properties. Our findings are fivefold. First, a total of 31 key phytochemicals were identified in both APC and UPC through UHPLC‐HRMS analysis, including compounds with known anti‐inflammatory properties such as apiin, APG, chlorogenic acid, bergapten, and phthalides (senkyunolide A and sedanolide). Second, these bioactive compounds were more abundant in APC than in UPC, suggesting its potential as a dietary source for health promotion. Third, quantitative analysis of 14 key phytochemicals associated with anti‐inflammatory effects revealed that the amounts of 11 compounds, with the exception of ferulic acid, scopoletin, bergapten, and sedanolide, were significantly higher in APC. Fourth, APCE and UPCE exhibited significant anti‐inflammatory properties in LPS‐stimulated RAW 264.7 cells by lowering NO production, suppressing iNOS and COX‐2 mRNA expression, reducing TNF‐α, IL‐1β, and IL‐6 levels, and inhibiting PGE2 secretion. Fifth, APC demonstrated a stronger anti‐inflammatory effect than APG, underscoring its greater potential for inflammation modulation. Taken together, these findings highlight the promising anti‐inflammatory potential of celeriac, revealing that both its commonly used UPC and its often‐discarded APC are valuable resources. This is the first study to provide a comprehensive phytochemical analysis of all parts of celeriac and to demonstrate their anti‐inflammatory effects.

In this research, a collective total of 31 compounds were provisionally identified in APC and UPC. Heatmap analysis revealed distinct differences between APC and UPC, with most compounds showing higher concentrations in APC, except for 9 compounds. The phytochemical compositions and quantity in plants are known to vary depending on plant part (Zhang et al. 2019), which could explain observed differences in compounds in APC and UPC. Moreover, phytochemicals can interact synergistically to produce more effective and beneficial health outcomes than individual phytochemicals (Zhang et al. 2019). Therefore, the observation that more compounds exhibited higher peaks in APC suggested its greater potential for delivering health benefits. In contrast, UPC exhibited higher peaks for hydroxycoumarins such as esculin and scopoletin. These compounds, as secondary metabolites in plants, can suppress pests or pathogens and exhibit antifungal and insecticidal activities (Li et al. 2014).

Celeriac is known to contain pharmacologically important plant‐based compounds, and it is recognized for its therapeutic properties. However, despite this recognition, research on its comprehensive phytochemical profile and specific bioactivities remains limited. Among the few studies available, some have identified various bioactive compounds, including phenols, flavonoids, and coumarins, in both the tubers and leaves of celeriac, highlighting their potential pharmacological effects (Popova et al. 2014; Yao et al. 2010). Building on this existing knowledge, the present study provides a more detailed characterization of the primary phytochemicals in both APC and UPC, contributing to a deeper understanding of their functional properties. In particular, a total of 14 compounds were quantified for their potential anti‐inflammatory effects and health benefits, including 5 hydroxycinnamic acids, 1 hydroxycoumarin, 1 flavonol, 2 flavones, 3 furanocoumarins, and 2 phthalides. Among hydroxycinnamic acids, 5‐feruloylquinic acid exhibited the highest concentration at 109.30 mg/kg, which was approximately 4.9 times higher in APC than in UPC. Similarly, chlorogenic acid, cryptochlorogenic acid, and 4‐coumaroyl quinic acid were found to be 5–11 times more abundant in APC. These findings suggest a significant difference in phytochemical composition between APC and UPC.

Chlorogenic acid has garnered significant attention for its pharmacological attributes and antioxidants, which include antitumor and anti‐inflammatory properties (Naveed et al. 2018; Wang et al. 2022). Studies have shown that cryptochlorogenic acid possesses antidiabetic, anti‐inflammatory, and dietary supplement properties (Bai et al. 2024; Zhao et al. 2020; Zhou 2020).

Scopoletin, a hydroxycoumarin, showed a significantly higher (approximately 4 times higher) concentration in UPC (28.90 mg/kg) than in APC (7.41 mg/kg). According to a study on celeriac by‐products, the scopoletin recovery rate was 6.9 μg/g (Motti et al. 2024). Comparing this to the present study, APC demonstrated a slightly higher scopoletin content, which was approximately 1.07 times higher, confirming a similar concentration level. Scopoletin has been studied as a possible treatment option for various conditions including antimicrobial, anticancer, liver diseases, diabetes, neurodegenerative disorders, and mental illnesses (Gao et al. 2024). Considering that APC is currently discarded as food waste and contains a higher amount of scopoletin compared to levels reported in a previous study on celery by‐products, it holds significant potential as a valuable resource for disease prevention and health promotion, especially given the limited phytochemical data available for celeriac.

Apiin, a flavone, was found in APC at a concentration of 3232.27 mg/kg, approximately 4.6 times higher than that in UPC, while APG was present in APC at 1.63 mg/kg, approximately 16 times higher than that in UPC. APG derived from the Apiaceae family, particularly the genus *Apium* (Sung et al. 2016), is recognized as a functional compound with potent anti‐inflammatory, antioxidant, and antitumor activities with therapeutic potential against various diseases (Kashyap et al. 2018; Mushtaq et al. 2023; Sung et al. 2016). APG is primarily found in the form of flavone glycosides, particularly apiin (apigenin‐7‐O‐apiosylglucoside), in celery leaves (Borges et al. 2022). Non‐glycosylated APG has high bioavailability due to its easier absorption across the intestinal mucosa (Meyer et al. 2006), whereas apiin has a lower bioavailability due to its resistance to β‐glucosidase, making it more difficult to be metabolized (Németh et al. 2003). In this study, apiin was found at a much higher concentration than APG, consistent with previous findings showing that APG content in celery varies by plant part, with leaves and flowers containing the highest levels, while petioles contain the lowest levels (Yan et al. 2014). APG has been shown to offer numerous health benefits, including significant anti‐inflammatory effects by decreasing inflammatory markers and enzymes and by suppressing pro‐inflammatory cytokine production in LPS‐treated groups (Park et al. 2020; Singh et al. 2023). Furthermore, combining APG with other natural compounds can enhance its anti‐inflammatory and antitumor activities through synergistic effects (Hong et al. 2021; Li et al. 2021). This suggests that apiin and APG found in APC and UPC could have a significant impact on their anti‐inflammatory properties and boost their overall health benefits.

Furanocoumarins are produced by plants such as those belonging to Apiaceae and Rutaceae. They contribute to the defense mechanisms of plants against insect, bacterial, and fungal predators. They are known to exhibit antimicrobial and insecticidal activities, and extracted furanocoumarins are widely used as antimicrobial agents in agricultural products (Fracarolli et al. 2016; Melough et al. 2018). Among furanocoumarins, bergapten was the most abundant, with a concentration of 80.66 mg/kg in APC and 91.05 mg/kg in UPC. It has been reported that bergapten can improve memory and pathological changes induced by scopolamine, increase brain cholinergic levels, and serve as a therapeutic agent for preventing neurodegeneration associated with Alzheimer's disease, and it has shown anticancer effects and potential for treating diabetes‐induced osteoporosis (Liang et al. 2021; Quetglas‐Llabrés et al. 2022). Moreover, bergapten possesses the capability to inhibit the synthesis of TNF‐α and IL‐6 induced by LPS stimulation, as well as the activation of NO, COX‐2, and PGE_2_ (Bose et al. 2011; Zhou et al. 2017). These results indicate that bergapten contained in APC and UPC is likely to contribute to the anti‐inflammatory effects observed in this study.

Phthalides are a specific category of compounds that are naturally found in a range of significant medicinal plants. They are recognized as being present in *Apium graveolens* (Donkor et al. 2016; Grube et al. 2019). Research has shown various diverse pharmacological activities, including suppression of osteoarthritis, amelioration of atherosclerosis, and anti‐inflammatory effects (Lei et al. 2019; Shao et al. 2022; Yang et al. 2021). Senkyunolide A was detected at 15,194.26 mg/kg in APC and 1145.04 mg/kg in UPC. Thus, the concentration of senkyunolide A in APC is approximately 13 times higher than that in UPC, suggesting that APC has significant potential as a functional material for health promotion. Sedanolide has been identified as having cytoprotective properties and the capacity to suppress the proliferation of cancer cells (Hsieh et al. 2015; Tabei et al. 2023). It was found at 7.96 mg/kg in APC and 33.00 mg/kg in UPC. Compared to its concentration of 34.2 μg/g in a celery by‐product extract reported in a previous study (Motti et al. 2024), APC showed approximately 4.2 times lower concentrations of sedanolide while UPC showed a similar level. These results suggest that utilizing both APC and UPC, which are currently discarded as food waste, could enhance resource efficiency and provide economic benefits when used together.

Celeriac contains a variety of phytochemicals, vitamins, and minerals, highlighting its significant potential as a health‐promoting food source. Total phenolic and flavonoid content in APC was higher than that in UPC, making APC a noteworthy source of antioxidant compounds (Popova et al. 2014). These compounds may provide advantages in terms of health by exhibiting antioxidant and anti‐inflammatory properties, which can enhance the utilization potential of APC. However, studies investigating the anti‐inflammatory properties of APC remain scarce.

APG, a flavonoid abundant in celery, parsley, and related plants, is recognized for its powerful inflammation‐reducing and antioxidant pharmacological effects (Park et al. 2020; Salehi et al. 2019). This research confirmed the presence of APG in both APC and UPC (Tables 2 and 3). Earlier research has shown that APG, found in celery, inhibits the production of NO induced (Che et al. 2020; Mencherini et al. 2007).

LPS‐stimulated RAW 264.7 cells serve as a prevalent in vitro model to study inflammatory responses such as the production of NO and PGE_2_. They are often employed to screen anti‐inflammatory agents and investigate action mechanisms of anti‐inflammatory drugs (Pi et al. 2014; Ryu et al. 2017). Inflammation serves as the immune system's primary line of defense against a range of external pathogens (Medzhitov 2008). Although NO is crucial for immune defense and inflammatory responses, excessive and prolonged production of this molecule can result in organ damage and play a role in the progression of severe chronic inflammatory diseases (Kolios et al. 2004; Korotkova et al. 2005). Furthermore, NO has the capacity to enhance the generation of cytokines that trigger inflammation, consequently augmenting the synthesis of nitric oxide. This interaction establishes a feedback loop that exacerbates the inflammatory response. Thus, inhibiting NO production is a key strategy for suppressing inflammation (Tian et al. 2021; Zhang et al. 2020).

The present research analyzed the cytotoxicity of APCE and UPCE, demonstrating that APCE showed no cytotoxicity up to 0.5 mg/mL and UPCE up to 0.13 mg/mL. A prior study has demonstrated that *S. ceratophylla* roots possess higher cytotoxicity than their aerial parts, suggesting that phytochemical compositions of plant parts such as roots, stems, and leaves can influence their cytotoxicity (Uysal et al. 2021). APCE and UPCE strongly inhibited NO production and iNOS mRNA expression (Figure 4). The results indicate that APCE and UPCE effectively inhibit LPS‐induced NO production and iNOS mRNA expression in a dose‐dependent manner, highlighting their potential for mitigating inflammatory responses. Additionally, they suppressed the expression of COX‐2 mRNA and secretion of PGE_2_ (Figure 5). Notably, APC extract at 0.5 mg/mL reduced NO production and iNOS mRNA expression to levels comparable to those in the APG‐treated group. Furthermore, COX‐2 mRNA expression was reduced to levels observed in the untreated LPS control group.

TNF‐α is a significant cytokine that modulates inflammatory responses and has a crucial function in both acute and chronic inflammatory conditions (Webster and Vucic 2020). Prolonged production of TNF‐α in individuals diagnosed with rheumatoid arthritis can lead to tissue inflammation and damage, ultimately resulting in bone and cartilage degradation (Mohd Zawawi et al. 2023). Notably, TNF‐ α has been observed to encourage the synthesis of IL‐6 and IL‐1β, enhancing inflammatory reactions and playing a role in the development of diseases, functional impairments, and mortality among the elderly (Tylutka et al. 2024).

LPS stimulation elicits a cascade leading to the activation of NF‐κB and the production of proinflammatory cytokines. Bioactive compounds (e.g., APG, bergapten) suppress IκBα phosphorylation, preventing NF‐κB nuclear translocation and subsequent transcription of pro‐inflammatory genes (iNOS, COX‐2, TNF‐ α, IL‐6) (Lau et al. 2021; Nicholas et al. 2007). This aligns with studies showing celery extracts reduce LPS‐induced NF‐κB activation in macrophages, thereby lowering inflammatory mediators (Shin et al. 2019).

APCE and UPCE exhibited anti‐inflammatory properties through the suppression of iNOS and COX‐2 expression, which resulted in reduced levels of NO and PGE_2_. Additionally, both extracts lowered the mRNA levels of TNF‐α, IL‐6, and IL‐1β. These findings strongly confirm that both parts of celeriac exhibit significant anti‐inflammatory properties, highlighting their potential in modulating inflammatory responses. Given these molecular mechanisms, the anti‐inflammatory potential of APC suggests promising applications in both the food and pharmaceutical industries. In functional food development, APC could serve as a source of natural anti‐inflammatory agents, potentially contributing to the prevention of chronic inflammatory conditions through dietary intake. In the pharmaceutical context, isolated bioactive compounds from APC may be further developed into therapeutic agents targeting inflammatory pathways. To fully translate these findings into practical applications, additional studies are needed, including investigations of bioavailability, safety, and clinical efficacy.

Chronic inflammatory diseases significantly contribute to global morbidity and mortality. A healthy lifestyle can reduce the likelihood of chronic inflammation and associated health problems (Arena et al. 2015). Anti‐inflammatory diets abundant in fruits and vegetables can provide phytochemicals with protective effects. They might help prevent chronic diseases and reduce inflammation (Zhang et al. 2019). The study identified 31 compounds with potential health benefits, including anticancer, antioxidant, and anti‐aging effects. This suggests that the entire celeriac, including discarded APC parts, could offer significant health‐promoting properties.

Despite the comprehensive phytochemical profiling and quantification presented in this study, some limitations remain. Notably, the biological efficacy of the identified phytochemicals has not been validated in vivo, leaving their functional relevance uncertain. Future studies should investigate the bioactivity of APC‐derived compounds using animal models and, eventually, clinical trials. In addition, evaluating the pharmacokinetics and bioavailability of key constituents will be crucial to assess their potential as therapeutic or nutraceutical agents. Furthermore, while the use of ANOVA and Duncan's multiple range test was appropriate for identifying statistically significant group differences, the analysis did not include effect size estimation or a priori power calculations. As a result, the interpretation of the practical significance and strength of the observed effects remains limited. Nonetheless, the current study provides valuable preliminary evidence that can inform subsequent, more robust investigations.

In this study, both APCE and UPCE demonstrated effectiveness in reducing NO production, iNOS and COX‐2 expression, and pro‐inflammatory cytokine mRNA levels. These findings underscore the anti‐inflammatory properties of celeriac, emphasizing its potential for developing therapeutic agents against inflammatory diseases or as a functional food ingredient. Furthermore, the results of this study highlight that APC, often discarded as waste, presents a promising resource for health promotion, with significant implications for waste reduction and enhancement of resource utilization. This understanding could offer valuable insights into their broader applications and effectiveness in managing inflammatory diseases.

## 5 Conclusions

In this study, we conducted a comprehensive analysis of the phytochemical composition of both the aerial and underground parts of celeriac and found that the aerial parts, which are often discarded, are particularly rich in bioactive compounds. Both APCE and UPCE exhibited anti‐inflammatory activity by downregulating key inflammatory mediators. These findings suggest that the aerial parts of celeriac may serve as a sustainable source of anti‐inflammatory agents, with potential applications in the development of functional foods or therapeutic products. By repurposing agricultural by‐products, this research supports both health promotion and environmental sustainability. Further studies investigating the bioavailability, mechanisms of action, and clinical relevance of APC and UPC phytochemicals are warranted to fully explore their utility in the prevention and treatment of inflammatory conditions.

## Author Contributions

**Sang A. Kwak:** conceptualization (equal), data curation (equal), formal analysis (equal), investigation (equal), project administration (equal), software (equal), supervision (equal), visualization (equal), writing – original draft (equal), writing – review and editing (equal). **Jane J. Lee:** conceptualization (equal), formal analysis (equal), investigation (equal), supervision (equal), writing – review and editing (equal). **Ju Hong Park:** funding acquisition (equal), supervision (equal), writing – review and editing (equal). **Nami Joo:** writing – review and editing (equal).

## Conflicts of Interest

The authors declare no conflicts of interest.

## Supporting information


**Table S1:** Selected reaction monitoring parameters of phytochemical compositions.
**Table S2:** Validation parameters for the quantitative analysis of selected phytochemicals in APC and UPC.
**Table S3:** Effects of APCE and UPCE on the cell viability of RAW 264.7 cells.
**Figure S1:** Calibration curves of the 14 quantified phytochemicals used for quantitative analysis.
**Figure S2:** LC–MS/MS chromatogram of phytochemicals. (A) Standard mixture; (B) Aerial parts of celeriac; (C) Underground parts of celeriac.
**Figure S3:** Total ion chromatogram of methanolic extracts of celeriac in positive ionization mode. (A) Aerial parts of celeriac, (B) Underground parts of celeriac.
**Figure S4:** Total ion chromatogram of methanolic extracts of celeriac in negative ionization mode. (A) Aerial part of celeriac, (B) Underground part of celeriac.
**Figure S5:** Heat map showing the quantified concentrations of selected phytochemicals in the aerial and underground parts of celeriac.

## Data Availability

The data that support the findings of this study are available from the corresponding author upon reasonable request.
