# Supplementary material for: Phytochemical Profiling and Anti‐Inflammatory Effects of Aerial and Underground Parts of Apium graveolens var. rapaceum (Celeriac): Potential Health Benefits of Discarded Aerial Parts
Source: Food Sci Nutr. 2025 Oct 3;13(10):e71011. doi: 10.1002/fsn3.71011 (PMC12491856; doi:10.1002/fsn3.71011)
Supplement: Supplementary file 1 — Table S1: Selected reaction monitoring parameters of phytochemical compositions. Table S2: Validation parameters for the quantitative analysis of selected phytochemicals in APC and UPC. Table S3: Effects of APCE and UPCE on the cell viability of RAW 264.7 cells. Figure S1: Calibration curves of the 14 quantified phytochemicals used for quantitative analysis. Figure S2: LC–MS/MS chromatogram of phytochemicals. (A) Standard mixture; (B) Aerial parts of celeriac; (C) Underground parts of celeriac. Figure S3: Total ion chromatogram of methanolic extracts of celeriac in positive ionization mode. (A) Aerial parts of celeriac, (B) Underground parts of celeriac. Figure S4: Total ion chromatogram of methanolic extracts of celeriac in negative ionization mode. (A) Aerial part of celeriac, (B) Underground part of celeriac. Figure S5: Heat map showing the quantified concentrations of selected phytochemicals in the aerial and underground parts of celeriac. [file FSN3-13-e71011-s001.docx]

Table S1. Selected reaction monitoring parameters of phytochemical compositions

| **#** | Compound | Adduct | Precursor Ion (*m/z*) | Quantifier Ion (*m/z*) [CE (V)] | Qualifier Ion 1 (*m/z*) [CE (V)] | Qualifier Ion 2 (*m/z*) [CE (V)] |
| --- | --- | --- | --- | --- | --- | --- |
|  | ***Hydroxycinnamic acids*** |  |  |  |  |  |
| 1 | Chlorogenic acid | [M−H]^−^ | 353.1 | 191.1 [16.4] | 179.1 [15.0] | 85.1 [40.1] |
| 2 | Cryptochlorogenic acid | [M−H]^−^ | 353.0 | 173.1 [16.1] | 179.1 [15.4] | 191.0 [22.8] |
| 3 | 4-Coumaroylquinic acid | [M−H]^−^ | 337.1 | 173.1 [14.8] | 93.1 [34.8] | 163.1 [16.8] |
| 4 | 5-Feruloylquinic acid | [M−H]− | 367.1 | 191.1 [15.8] | 173.1 [18.1] | 193.1 [19.8] |
| 5 | Ferulic acid | [M−H]^−^ | 192.9 | 134.0 [14.4] | 149.0 [16.5] | 192.9 [11.1] |
|  | ***Hydroxycoumarin*** | [M−H]^−^ | 191.0 | 176.0 [14.5] | 104.1 [24.8] |  |
| 1 | Scopoletin | [M−H]^−^ | 271.1 | 151.1 [17.6] | 119.1 [26.1] | 148.1 [21.0] |
|  | ***Flavonol*** |  |  |  |  |  |
| 1 | Kaempferol 3-sambubioside | [M−H]− | 579.2 | 284.1 [31.6] | 227.1 [54.7] | 255.1 [51.8] |
|  | ***Flavones*** |  |  |  |  |  |
| 1 | Apiin | [M−H]^−^ | 563.2 | 269.1 [35.0] | 413.1 [28.2] | 431.1 [24.3] |
| 2 | Apigenin | [M−H]^−^ | 269.1 | 117.1 [35.0] | 149.1 [24.3] | 151.1 [24.5] |
|  | ***Furanocoumarins*** |  |  |  |  |  |
| 1 | Psoralen | [M+H]^+^ | 187.0 | 131.1 [26.0] | 145.9 [8.5] | 166.5 [5.3] |
| 2 | Bergapten | [M+H]^+^ | 217.1 | 202.1 [21.2] | 139.0 [15.1] | 174.1 [28.8] |
| 3 | Imperatorin | [M+H]^+^ | 271.0 | 203.1 [11.7] | 147.0 [32.6] | 175.1 [24.8] |
|  | ***Phthalides*** |  |  |  |  |  |
| 1 | Senkyunolide A | [M+H]^+^ | 193.0 | 147.1 [12.0] | 91.1 [26.2] | 137.1 [13.7] |
| 2 | Sedanolide | [M−H]^−^ | 192.0 | 177.1 [14.4] | 105.0 [24.6] | 149.0 [21.4] |

Abbreviations: CE (V), Collision Energy (V).

Table S2. Validation parameters for the quantitative analysis of selected phytochemicals in APC and UPC

| **#** | Compound | Retention Time  (min) | Linear Range  (ng/mL) | R^2^ | LOD (ng/g) | LOQ (ng/g) | Accuracy (%) | Precision (%) |
| --- | --- | --- | --- | --- | --- | --- | --- | --- |
|  | ***Hydroxycinnamic acids*** |  |  |  |  |  |  |  |
| 1 | Chlorogenic acid | 3.89 | 1-1000 | 0.9999 | 0.07544 | 0.22859 | 0.0754 | 0.2286 |
| 2 | Cryptochlorogenic acid | 3.89 | 1-500 | 0.9999 | 0.05415 | 0.16408 | 0.0541 | 0.1641 |
| 3 | 4-Coumaroylquinic acid | 4.31 | 1-500 | 0.9998 | 0.07511 | 0.22760 | 0.0751 | 0.2276 |
| 4 | 5-Feruloylquinic acid | 4.50 | 1-500 | 0.9999 | 0.07092 | 0.21492 | 0.0709 | 0.2149 |
| 5 | Ferulic acid | 5.11 | 1-500 | 0.9998 | 0.07962 | 0.24126 | 0.1138 | 0.3448 |
|  | ***Hydroxycoumarin*** |  |  |  |  |  |  |  |
| 1 | Scopoletin | 5.06 | 1-500 | 0.9999 | 0.05196 | 0.15746 | 0.0520 | 0.1575 |
|  | ***Flavonol*** |  |  |  |  |  |  |  |
| 1 | Kaemprerol-3-sambubioside | 5.00 | 1-500 | 0.9999 | 0.10616 | 0.32168 | 0.0796 | 0.2413 |
|  | ***Flavones*** |  |  |  |  |  |  |  |
| 1 | Apiin | 5.41 | 1-500 | 0.9999 | 0.11379 | 0.34481 | 0.0987 | 0.2991 |
| 2 | Apigenin | 7.32 | 1-1000 | 0.9999 | 0.09870 | 0.29910 | 0.0898 | 0.2722 |
|  | ***Furanocoumarins*** |  |  |  |  |  |  |  |
| 1 | Psoralen | 7.06 | 1-500 | 0.9999 | 0.08152 | 0.24703 | 0.0815 | 0.2470 |
| 2 | Bergapten | 7.83 | 1-500 | 0.9999 | 0.08982 | 0.27217 | 0.1028 | 0.3114 |
| 3 | Imperatorin | 9.55 | 1-500 | 0.9998 | 0.10275 | 0.31137 | 0.0666 | 0.2019 |
|  | ***Phthalides*** |  |  |  |  |  |  |  |
| 1 | Senkyunolide A | 5.05 | 1-1000 | 0.9999 | 0.07501 | 0.22730 | 0.0750 | 0.2273 |
| 2 | Sedanolide | 9.03 | 1-500 | 0.9999 | 0.06663 | 0.20190 | 0.1062 | 0.3217 |

Abbreviations: APC, aerial parts of celeriac; LOD, limit of detection; LOQ, limit of quantification; UPC, underground parts of celeriac.

Table S3. Effects of APCE and UPCE on the cell viability of RAW 264.7 cells

| Control | 100±0.00^bBC^ | | | | | | | | | | |
| --- | --- | --- | --- | --- | --- | --- | --- | --- | --- | --- | --- |
| Concentration  (mg/mL) | 0.01 | 0.02 | 0.03 | 0.06 | 0.13 | 0.25 | 0.5 | 1 | SDS | F-value |  |
| APCE | 99.92±0.47^b^ | 101.44±3.30^b^ | 104.74±4.68^b^ | 108.37±8.47^ab^ | 112.46±9.08^ab^ | 119.49±12.90^a^ | 112.69±10.25^ab^ | 77.52±3.83^c^ | 9.13±2.67^d^ | 64.183^***^ |  |
| UPCE | 103.54±2.49^BC^ | 105.80±2.15^ABC^ | 109.73±2.88^A^ | 109.40±3.98^A^ | 102.11±2.44^BC^ | 65.21±1.46^D^ | 16.09±2.87^E^ | 10.94±3.03^F^ | 9.87±2.49^F^ | 896.624^***^ |  |

Cells were treated with increasing concentrations (0.01-1 mg/mL) of APCE and UPCE for 24 h.

Cell viability was measured using a WST-1 cell viability assay kit.

The control group refers to untreated cells without LPS stimulation.

Results are expressed as cell viability (% of control).

All data are presented as mean ± standard deviation (n = 3).

a-d, A-F Mean values with different letters are significantly different (p < 0.05) among groups as determined by Duncan’s multiple range test.

Abbreviations: APCE, aerial parts of celeriac extract; LPS, lipopolysaccharide; SDS, sodium dodecyl sulfate; UPCE, underground parts of celeriac extract.


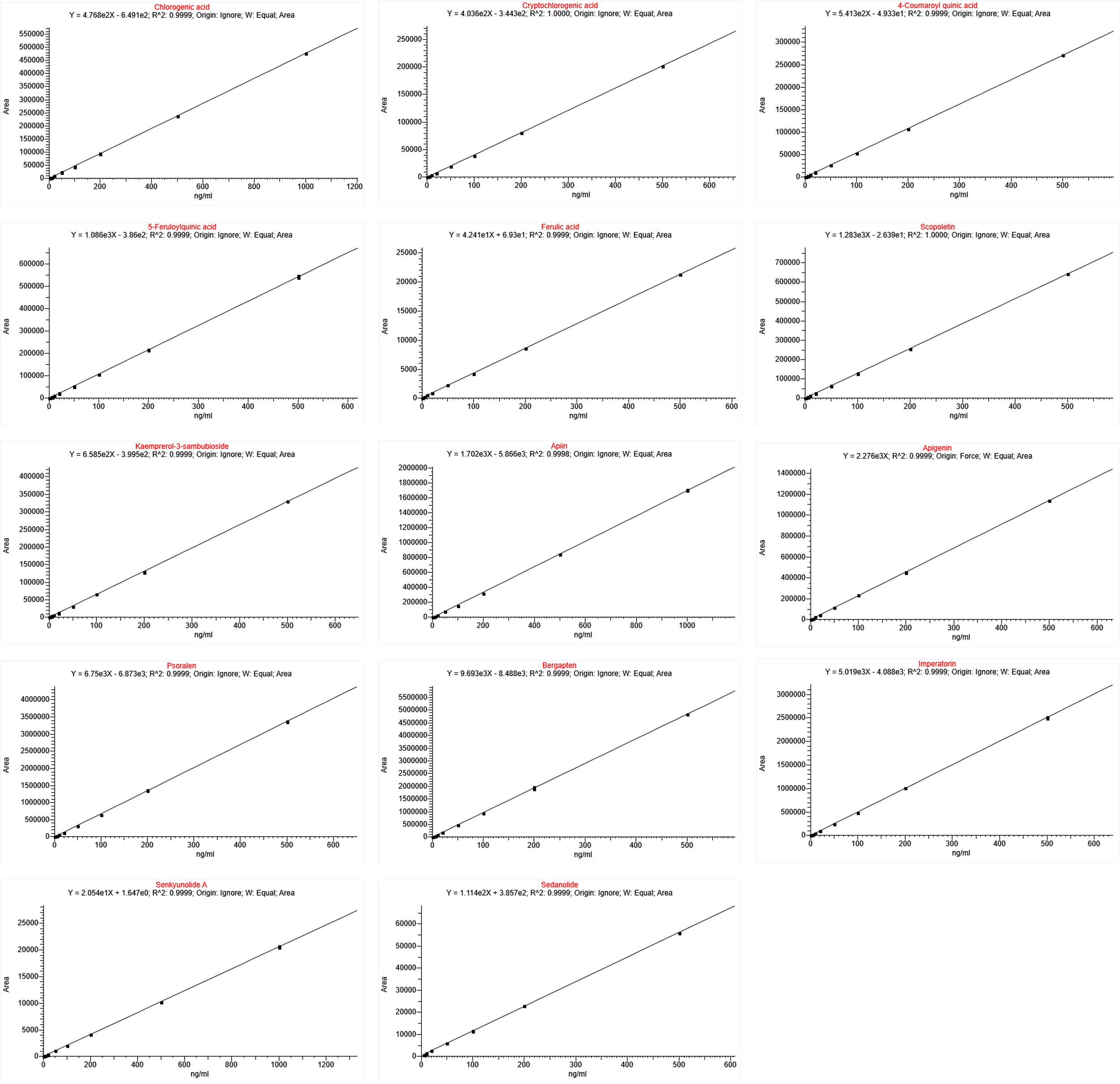


Figure S1. Calibration curves of the 14 quantified phytochemicals used for quantitative analysis

(A)


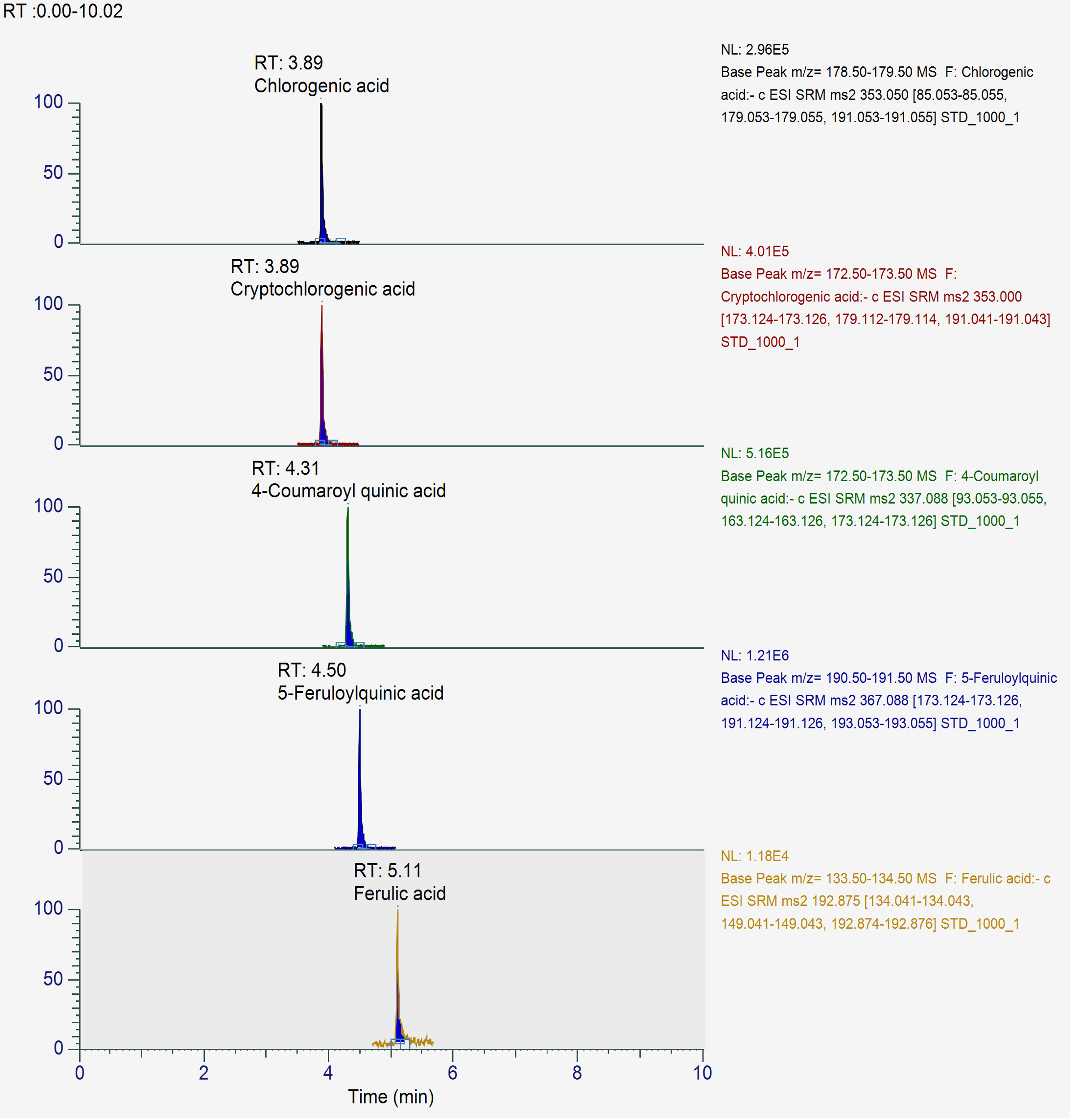


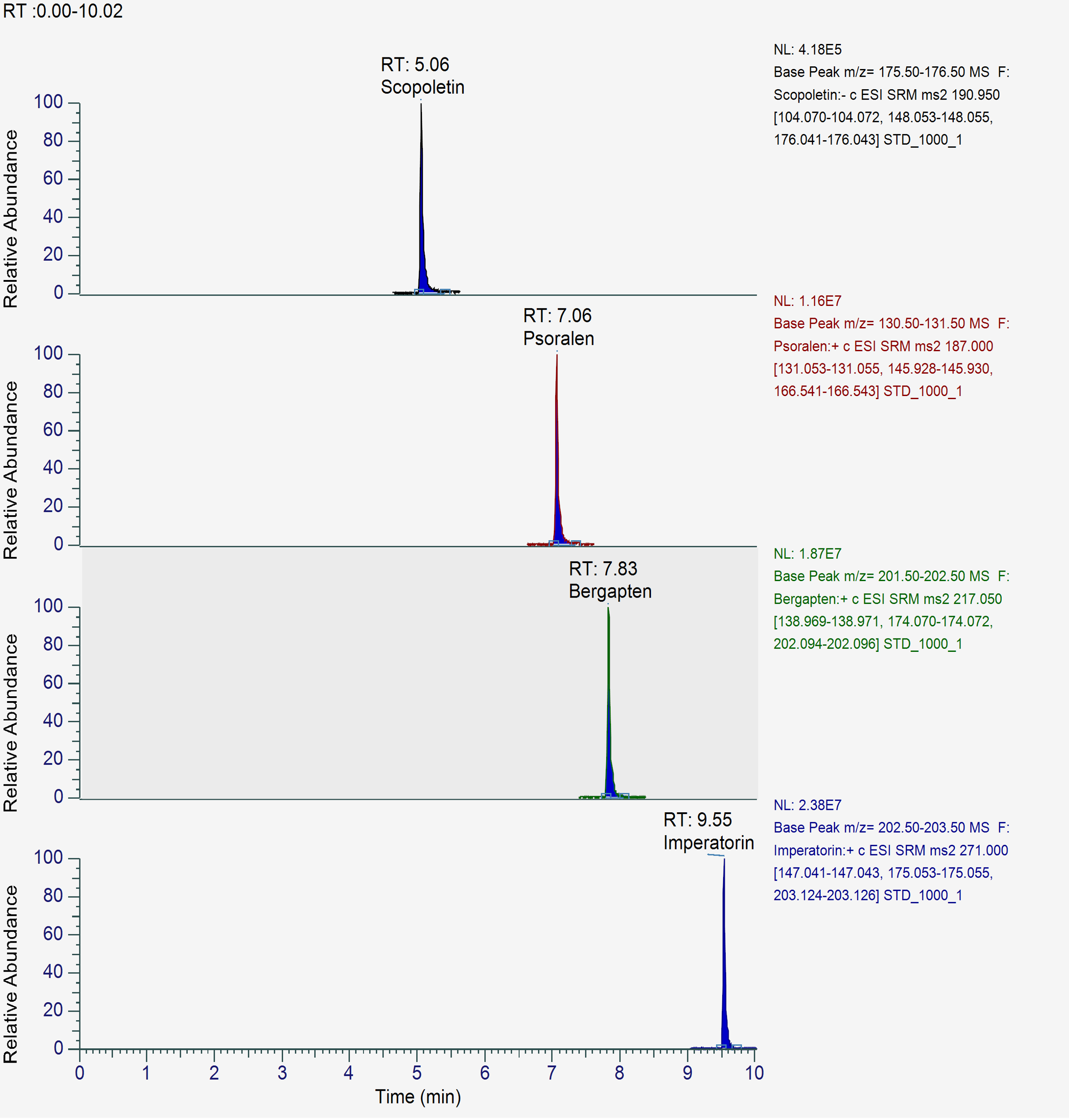


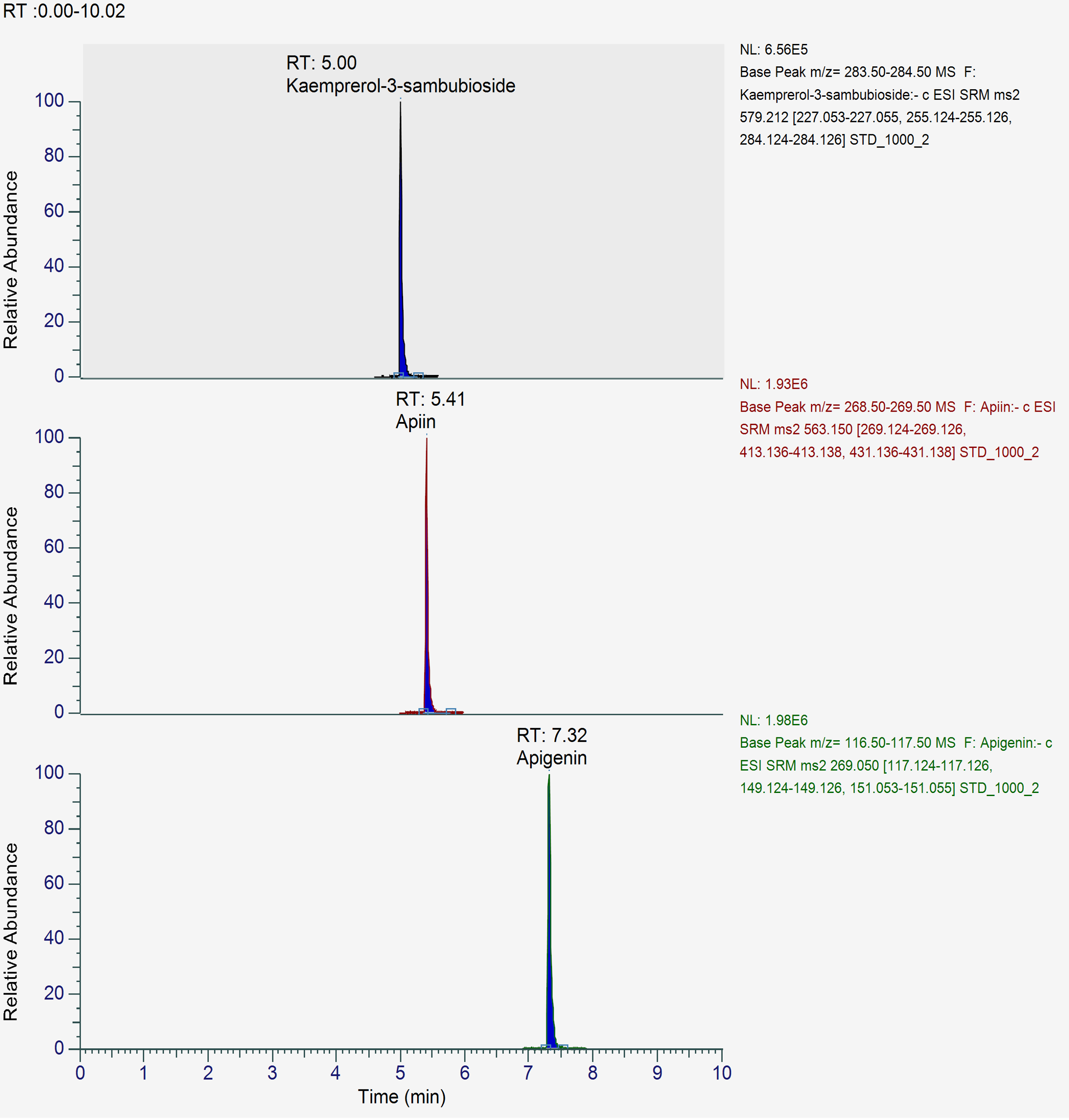


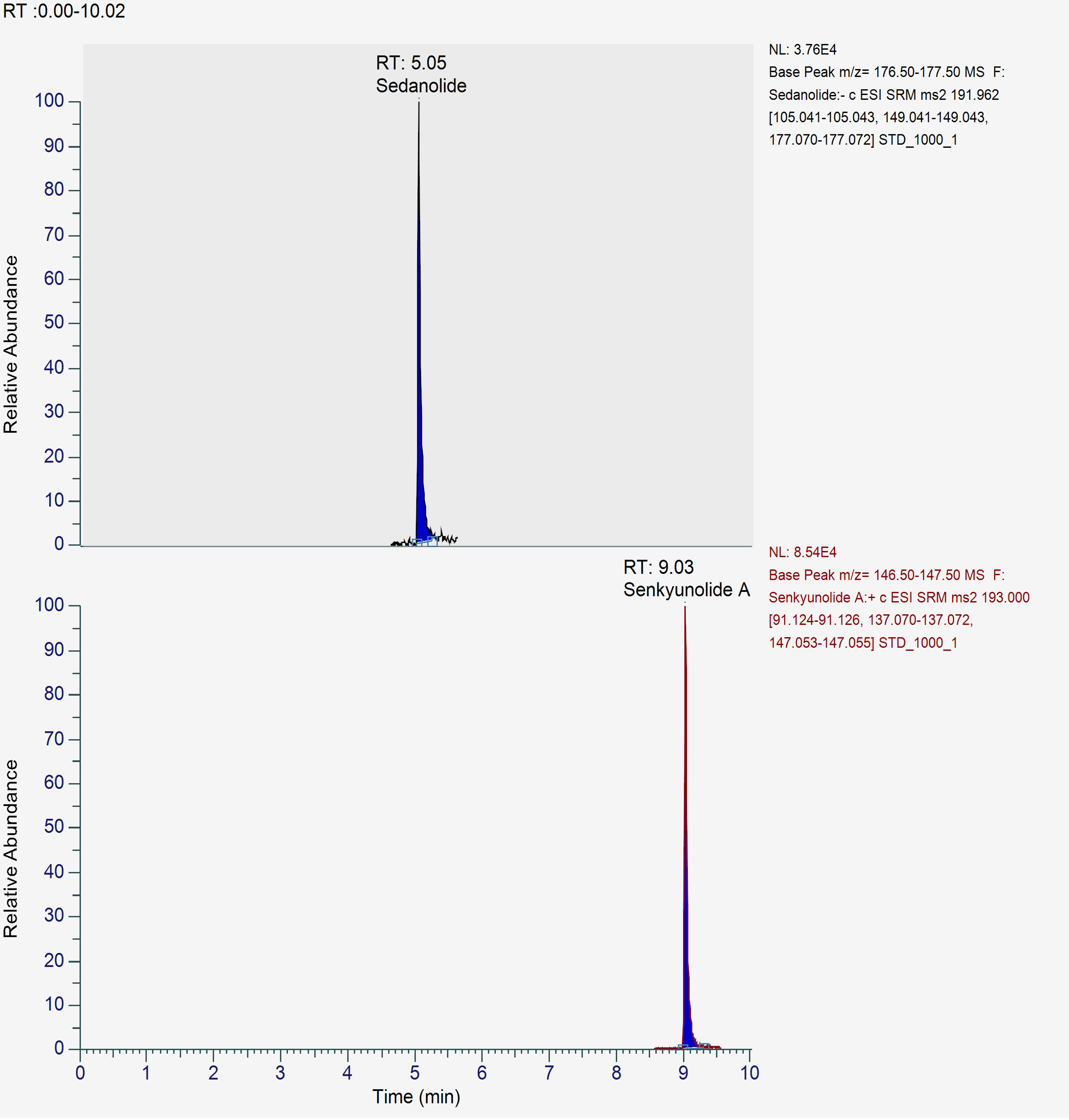


(B)


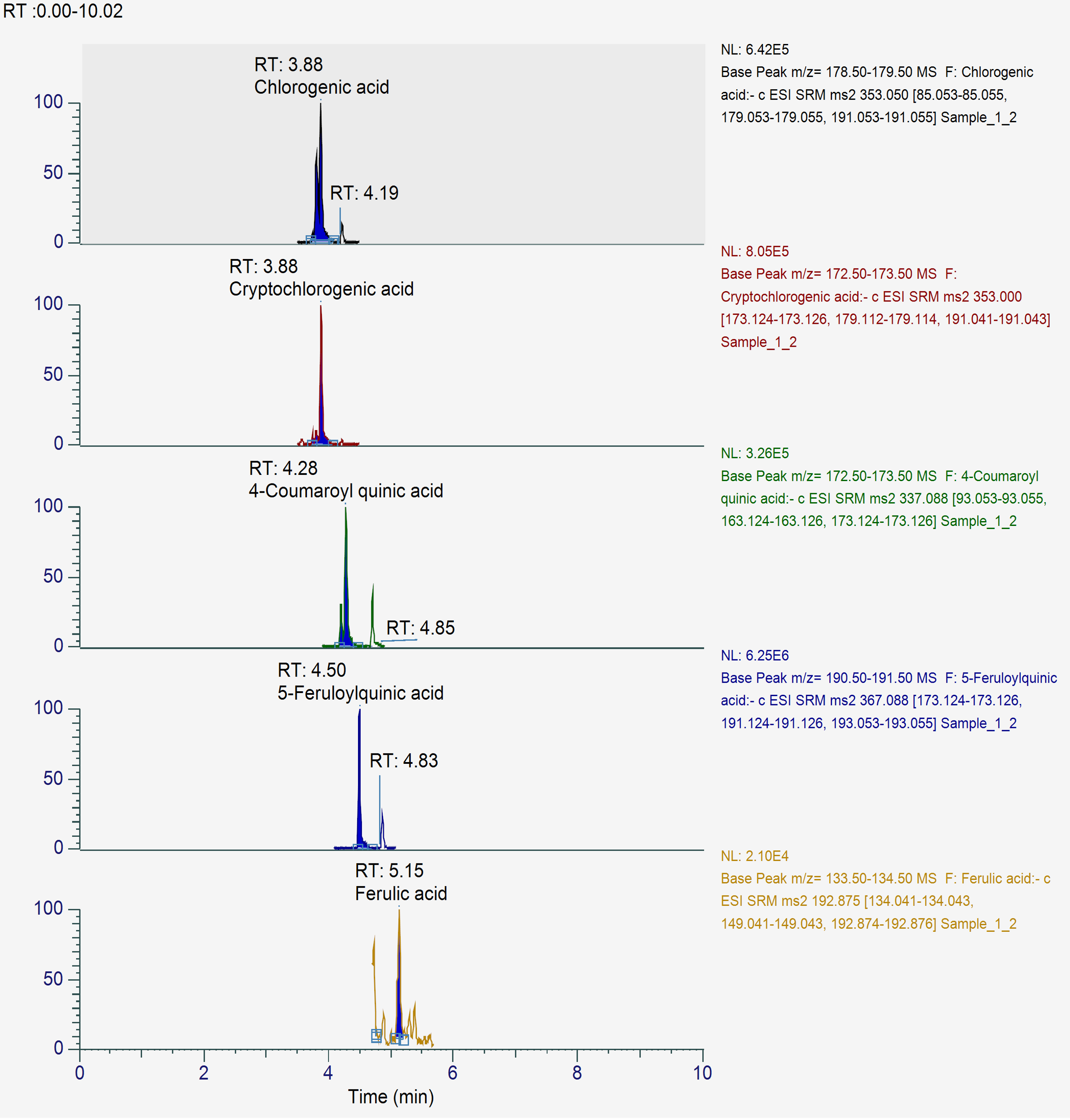


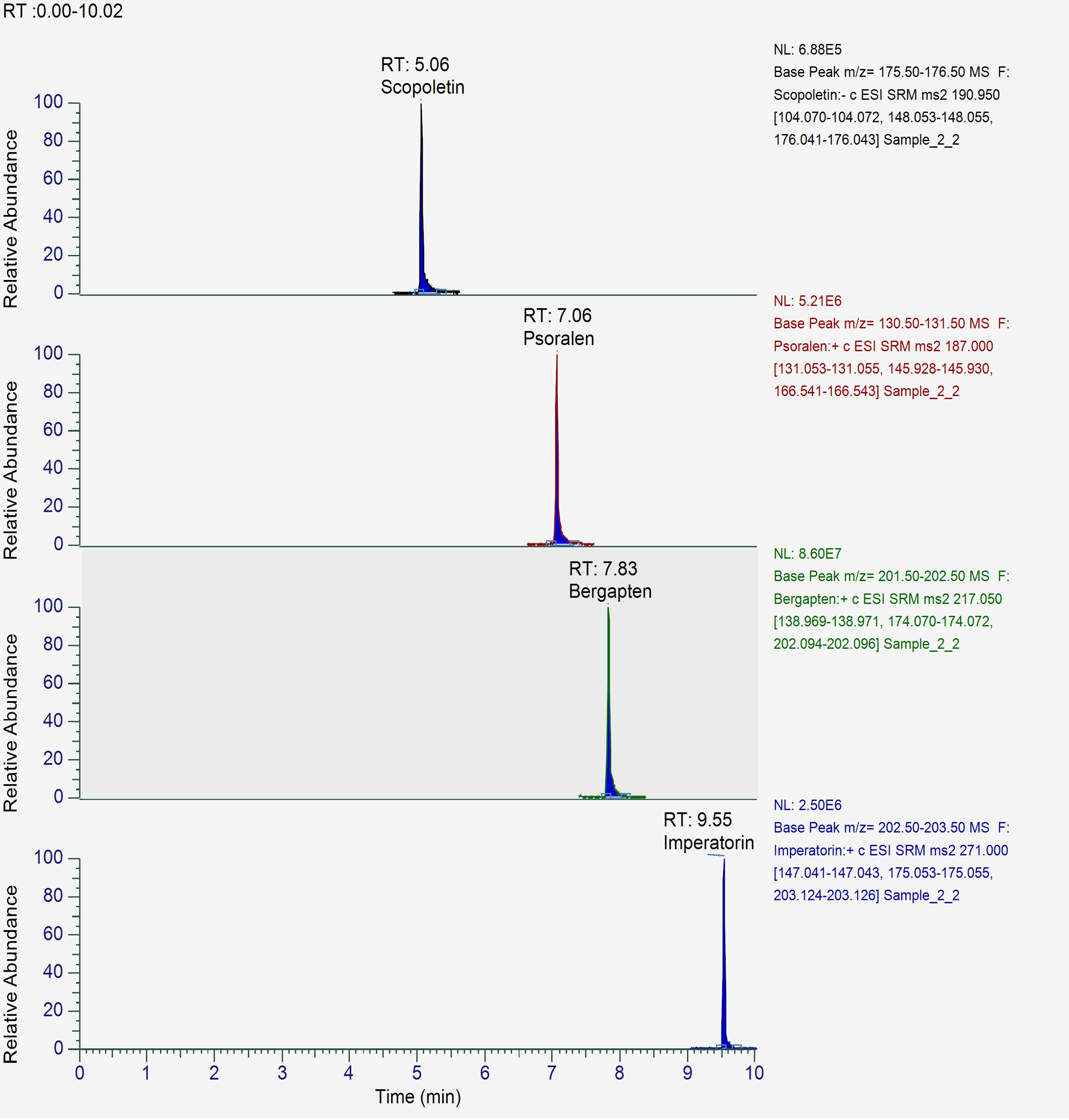


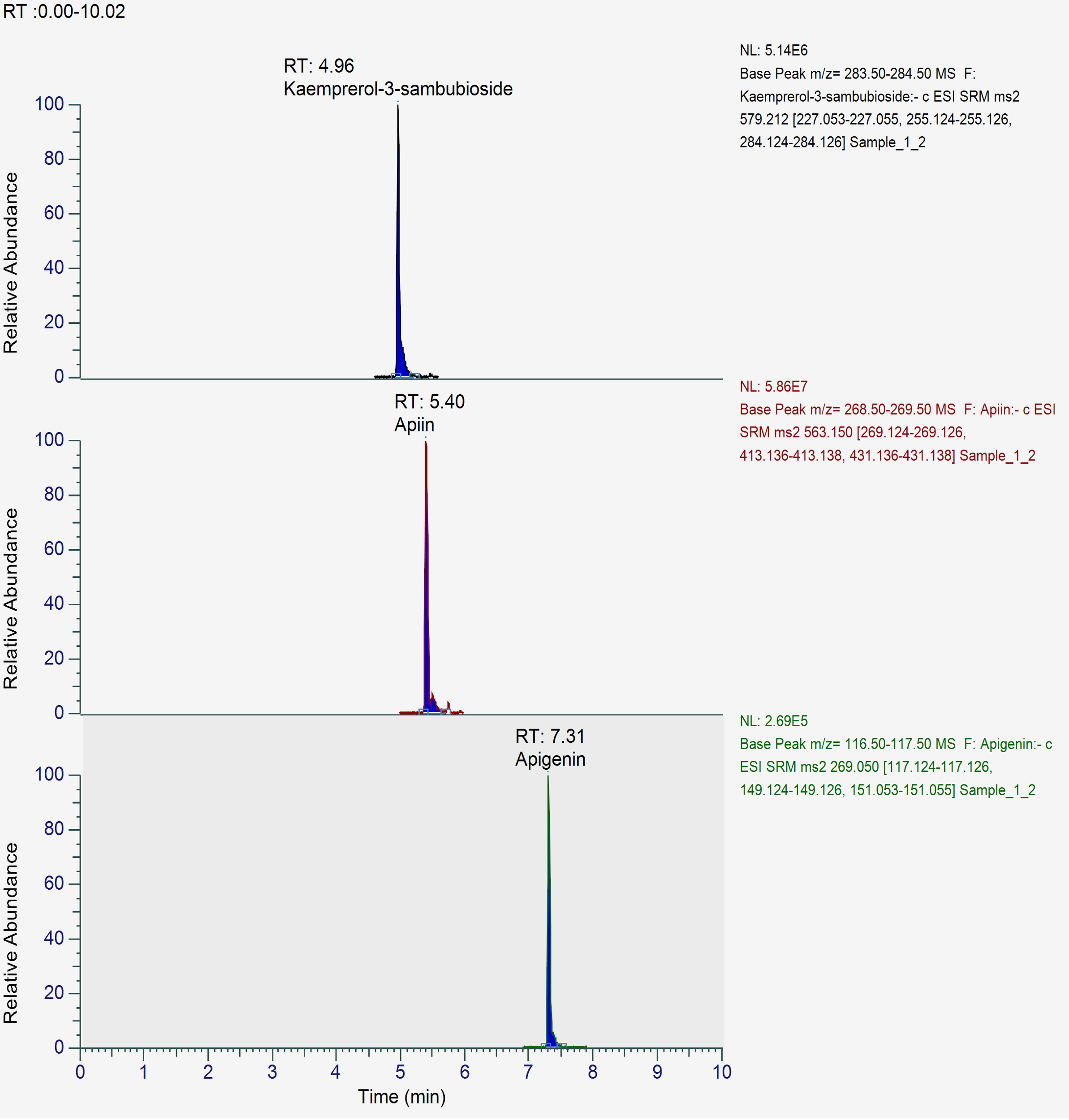


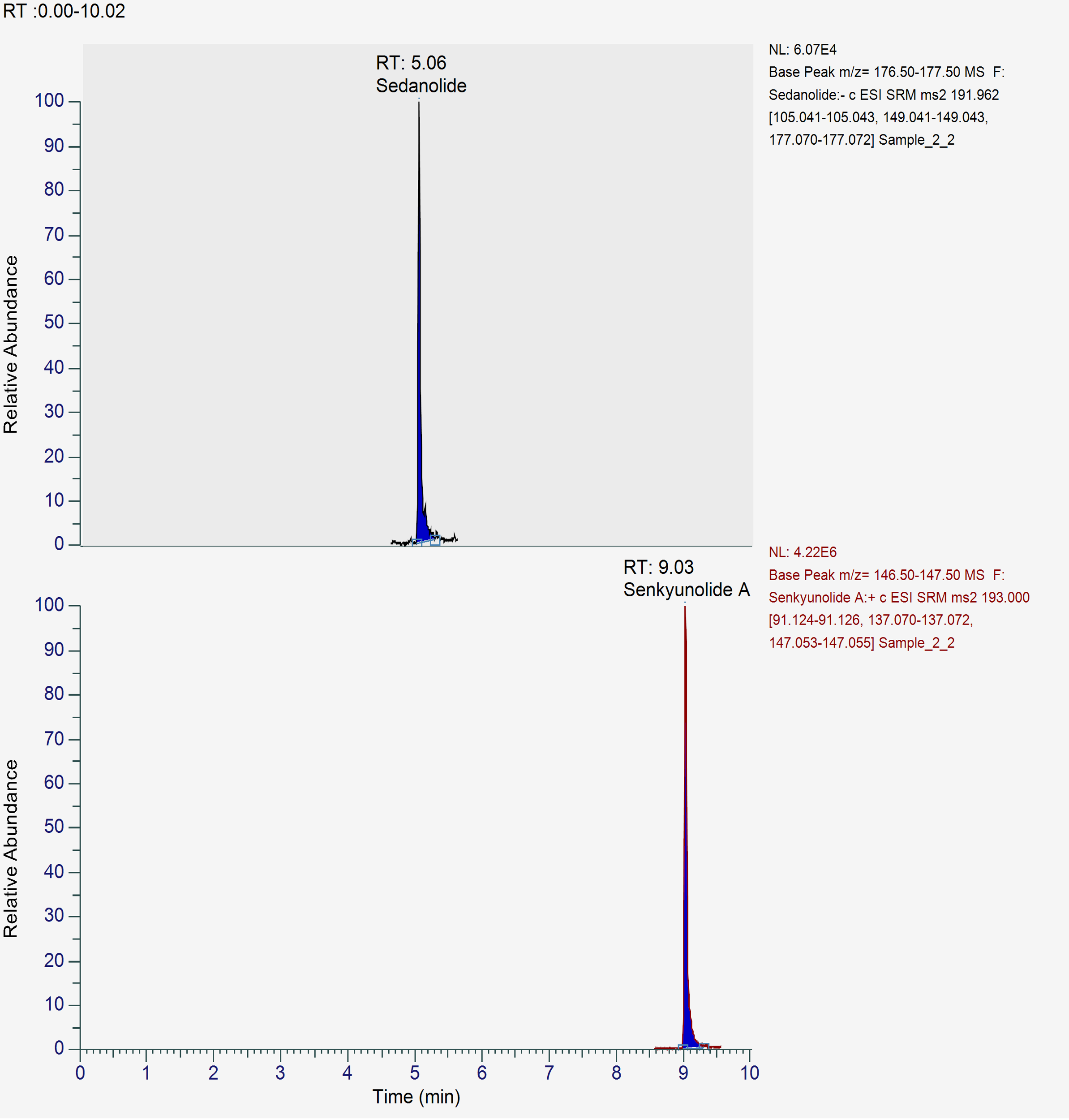


(C)


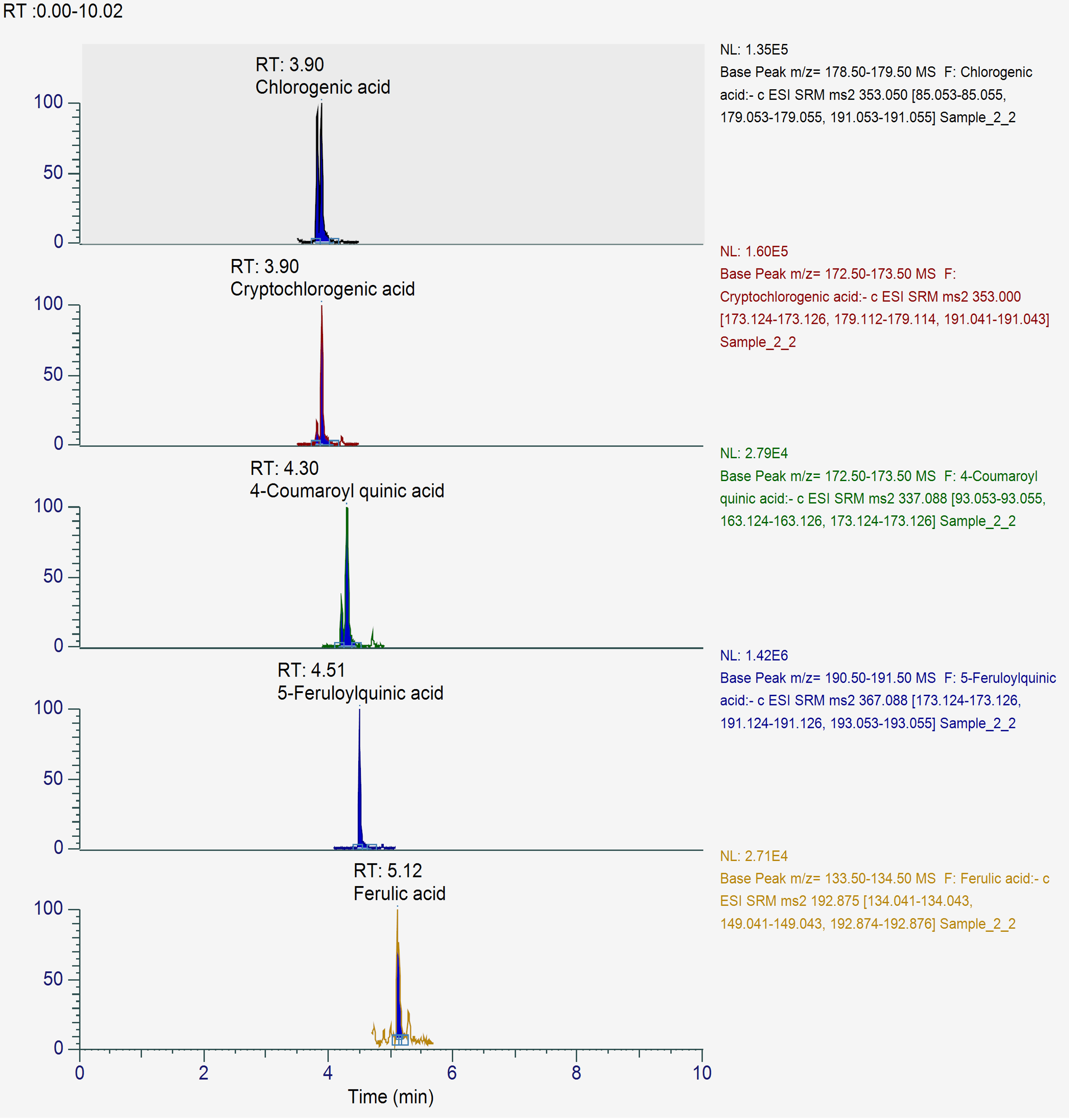


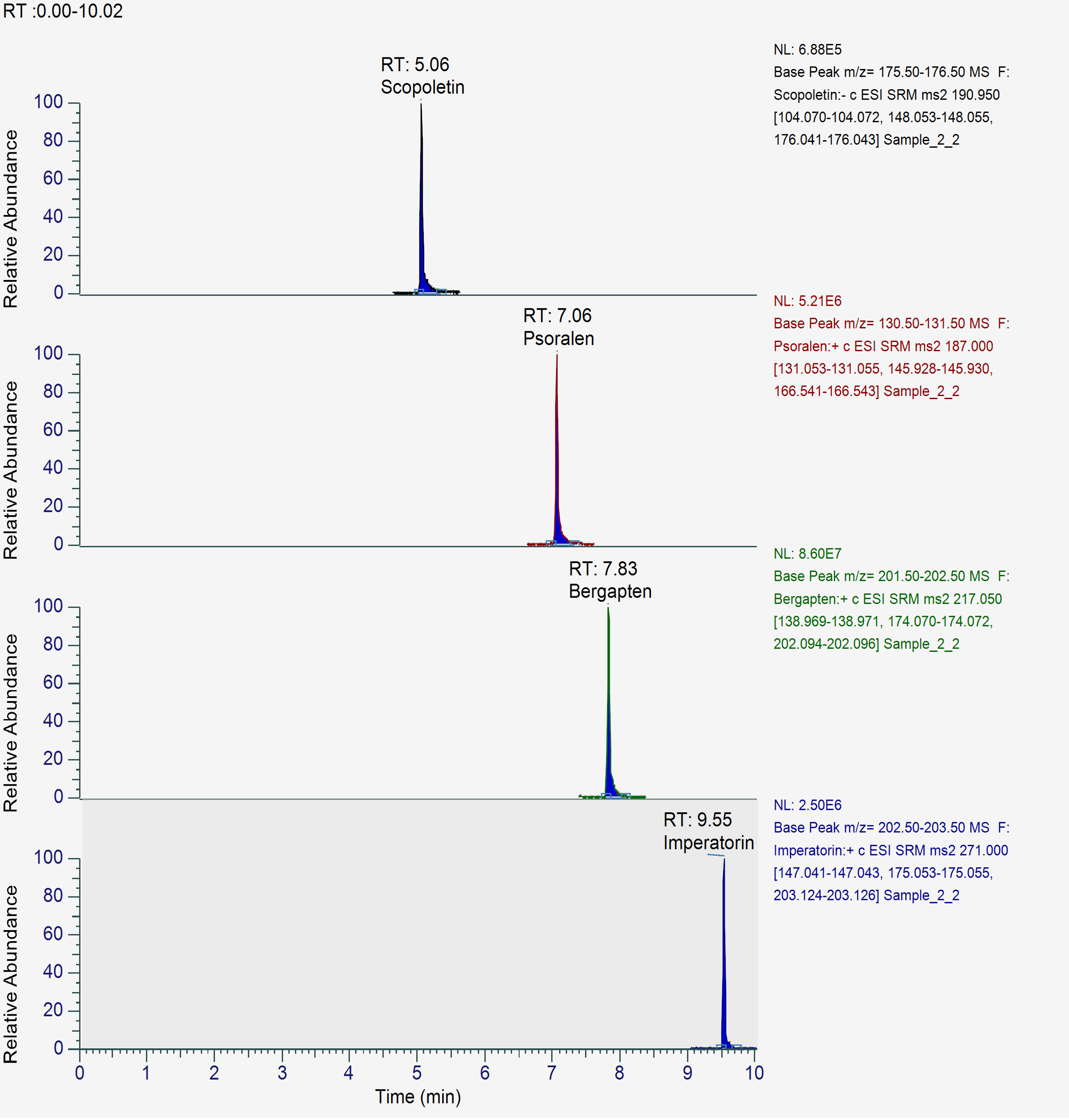


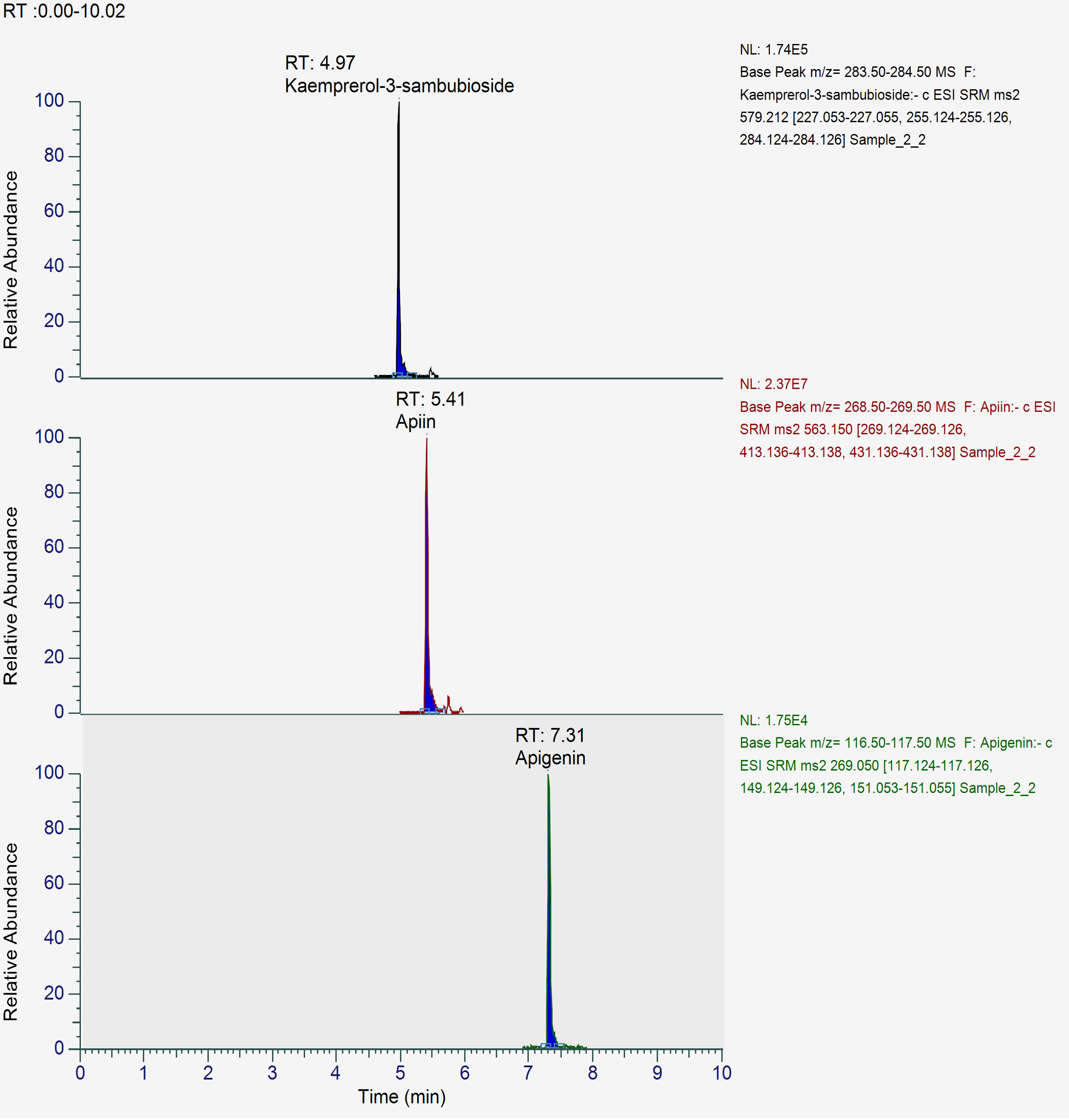


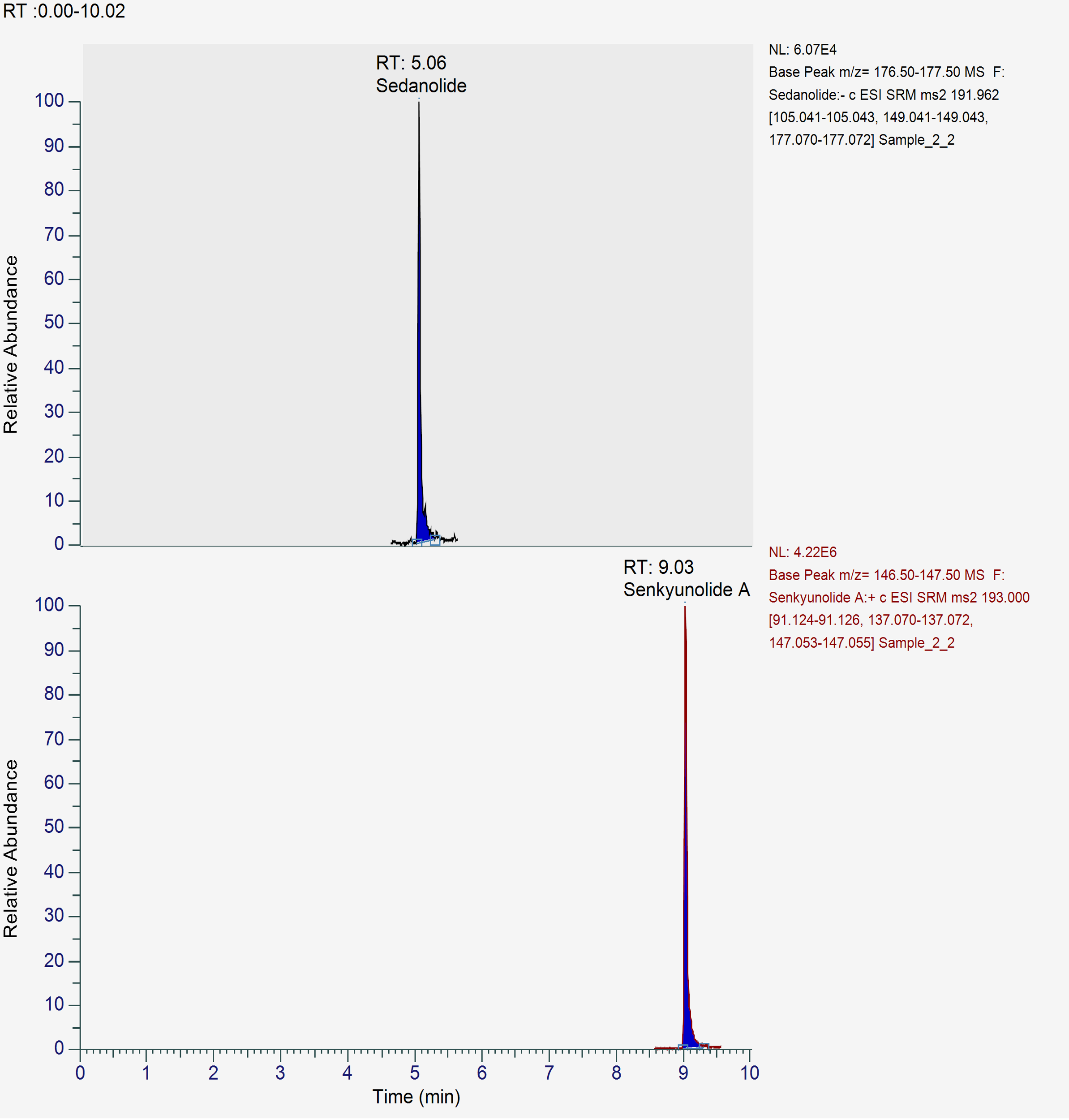


Figure S2. LC-MS/MS chromatogram of phytochemicals. (A) Standard mixture; (B) Aerial parts of celeriac; (C) Underground parts of celeriac.

Abbreviation: RT, retention time.

(A)


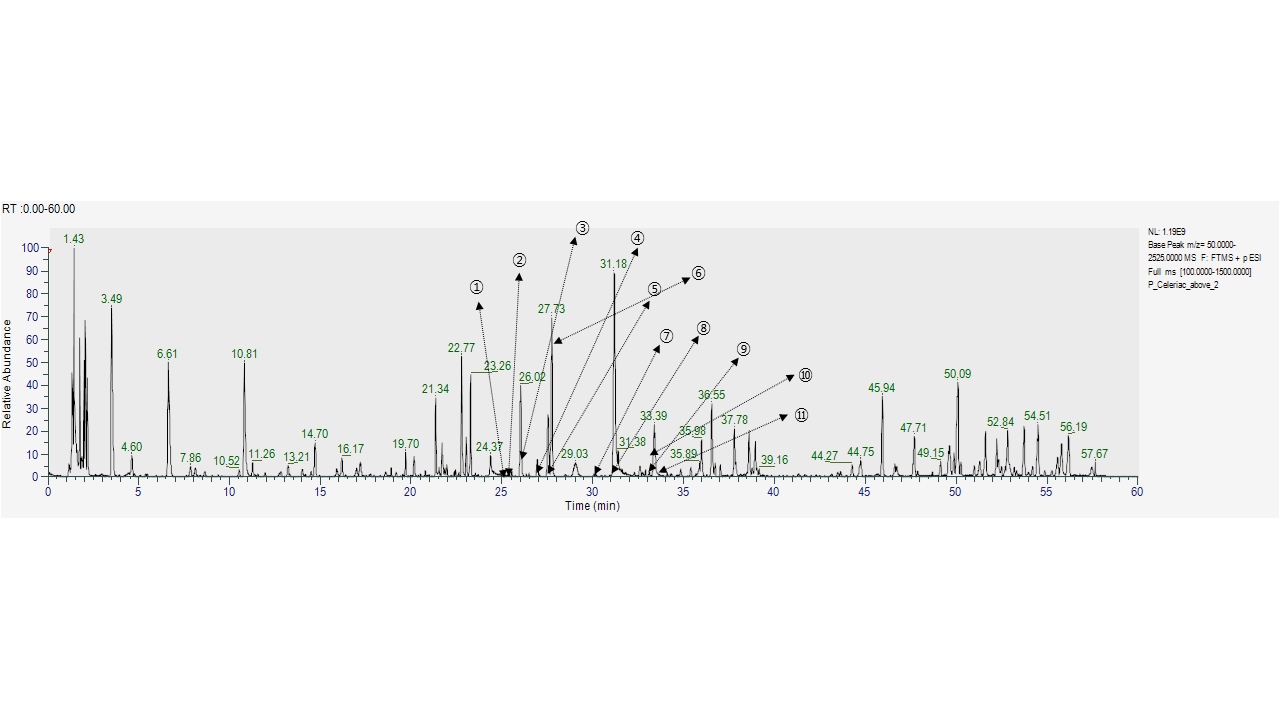


(B)


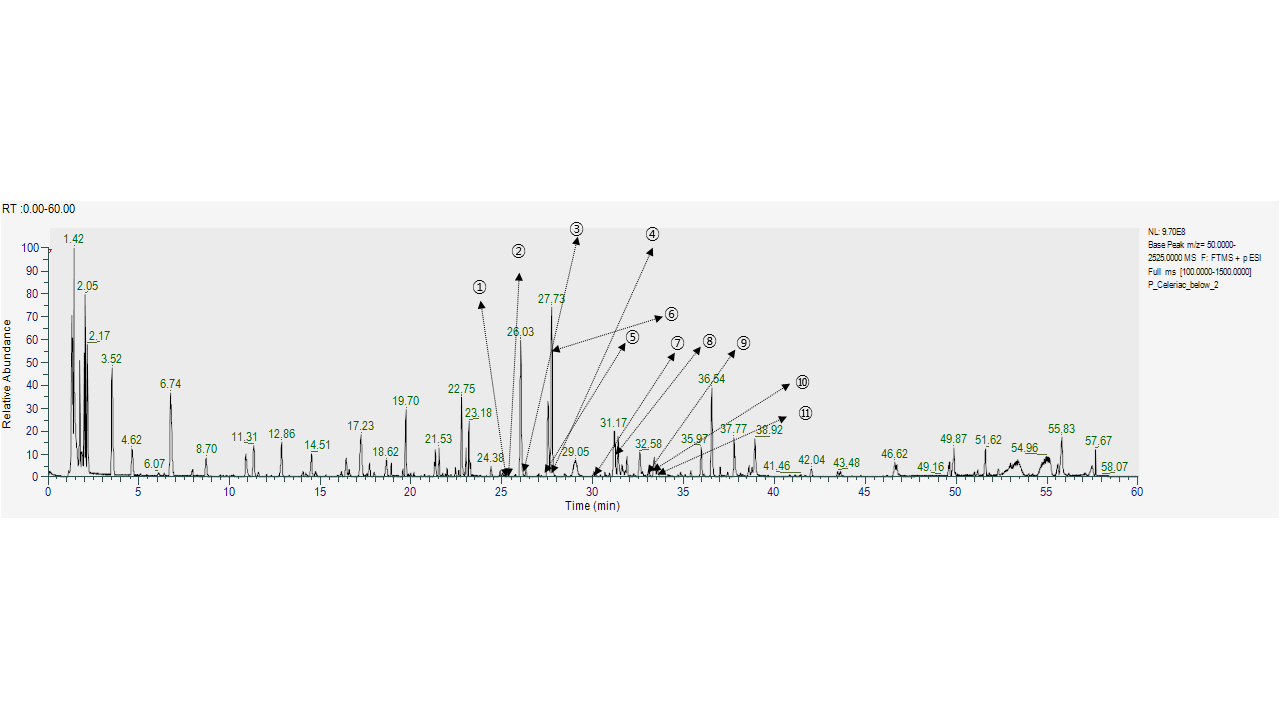


Figure S3. Total ion chromatogram of methanolic extracts of celeriac in positive ionization mode. (A) Aerial parts of celeriac, (B) Underground parts of celeriac.

①, Psoralen; ②, Fraxinol methyl ether; ③, Isobergaptene; ④, Citropten; ⑤, Bergapten; ⑥, Isopimpinellin; ⑦, Peucenin; ⑧, Senkyunolide A; ⑨, Imperatorin; ⑩, Sedanolide; ⑪, Ligustilide

Abbreviation: RT, retention time.

(A)


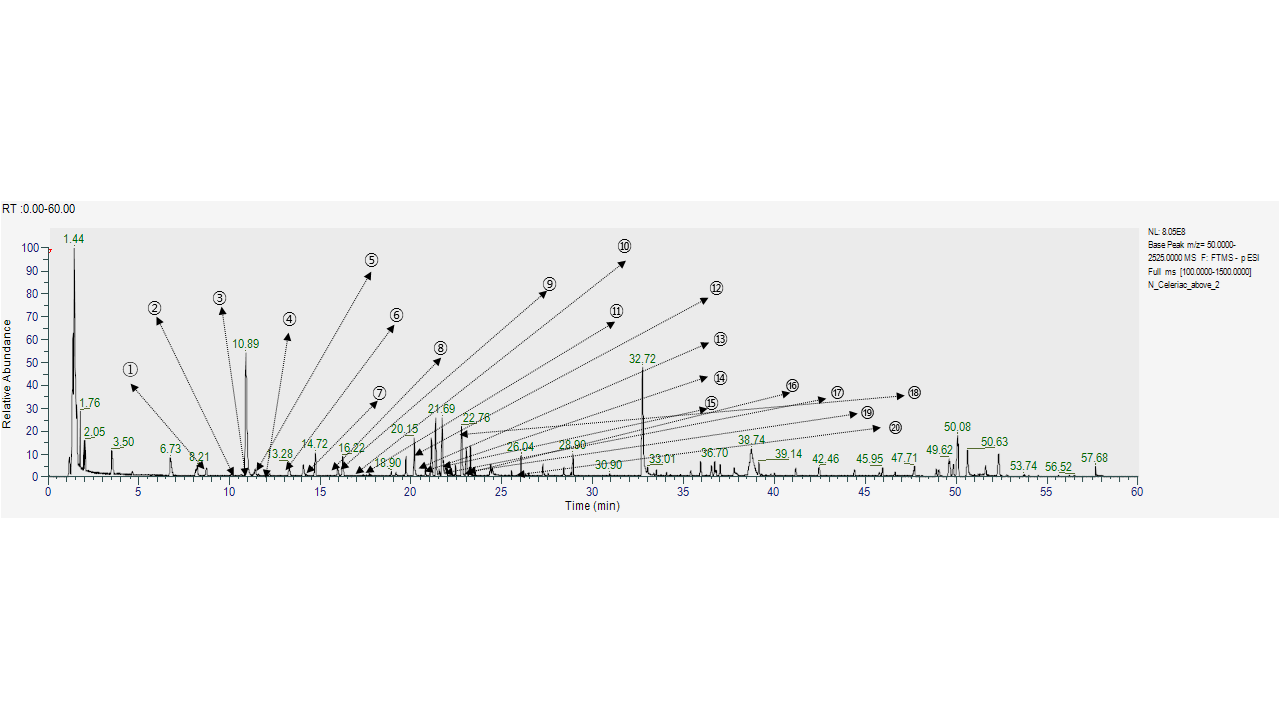


(B)


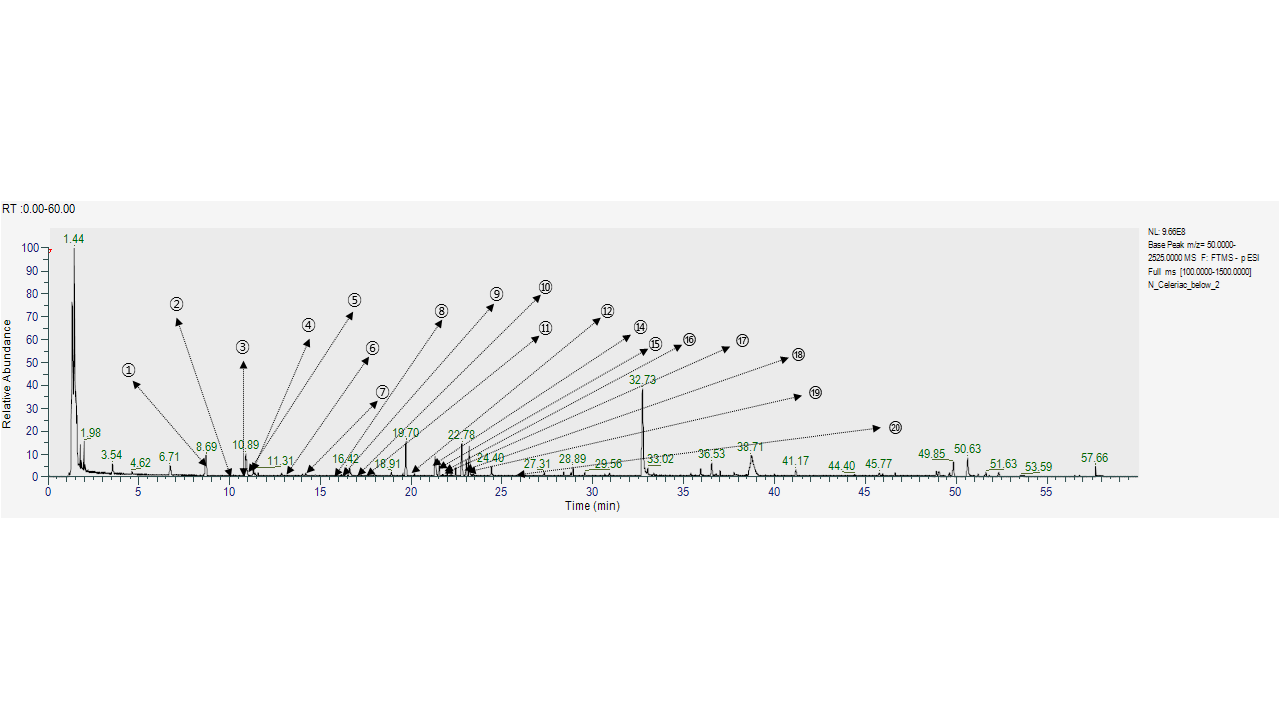


Figure S4. Total ion chromatogram of methanolic extracts of celeriac in negative ionization mode. (A) Aerial part of celeriac, (B) Underground part of celeriac.

①, Esculin; ②, Caffeic acid hexoside; ③, Chlorogenic acid; ④, Caffeic acid; ⑤, Cryptochlorogenic acid; ⑥, 1-Caffeoylquinic acid; ⑦, 4-Coumaroylquinic acid; $\mathbf{⑧,}$5-Feruloylquinic acid; ⑨, 1-Coumaroylquinic acid; ⑩, Scopoletin; ⑪, Ferulic acid; ⑫, kaempferol 3-sambubioside; ⑬, Cynaroside; ⑭ Apiin; ⑮, Diosmetin-7-O-arabinoglucoside; ⑯, Luteolin 7-O-(6''-malonylglucoside); ⑰, Diosmetin-7-O-glucoarabinoside; ⑱, 6''-Malonylapiin; ⑲, Diosmetin; ⑳, Apigenin

Abbreviation: RT, retention time.


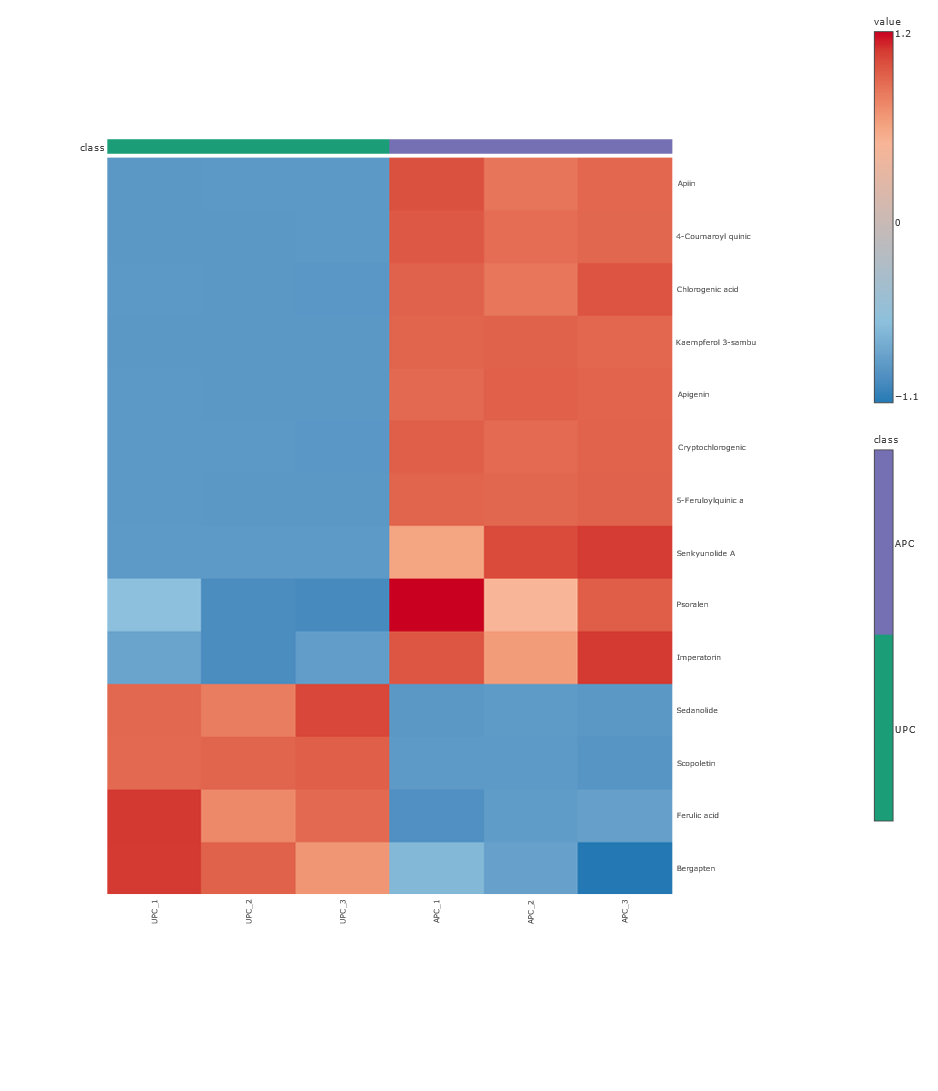
Figure S5. Heat map showing the quantified concentrations of selected phytochemicals in the aerial and underground parts of celeriac.

This heat map was constructed using the web-based MetaboAnalyst platform.

Abbreviations: APC, aerial parts of celeriac; UPC, underground parts of celeriac.
